# Systematic Characterization and Identification of Saikosaponins in Extracts From *Bupleurum marginatum var. stenophyllum* Using UPLC-PDA-Q/TOF-MS

**DOI:** 10.3389/fchem.2021.747987

**Published:** 2021-09-30

**Authors:** Wenxi Liu, Xianlong Cheng, Rong Kang, Yadan Wang, Xiaohan Guo, Wenguang Jing, Feng Wei, Shuangcheng Ma

**Affiliations:** ^1^ Chinese Academy of Medical Science and Peking Union Medical College, Beijing, China; ^2^ National Institutes for Food and Drug Control, National Medical Products Administration, Beijing, China

**Keywords:** saikosaponins, radix bupleuri, UPLC-PDA-Q/TOF-MS, *Bupleurum marginatum var. stenophyllum*, *Bupleurum chinense* DC, *Bupleurum marginatum* Wall. ex DC

## Abstract

Saikosaponins comprise a large group of chemical components present in the *Bupleurum* species that have attracted attention in the field of medicine because of their significant biological activities. Due to the high polarity, structural similarity, and the presence of several isomers of this class of components, their structural identification is extremely challenging. In this study, the mass spectrometric fragmentation pathways, UV spectral features, and chromatographic behavior of different types of saikosaponins were investigated using 24 standard substances. Saikosaponins containing carbonyl groups (C=O) in the aglycone produced fragment ions by loss of 30 Da, and in addition, type IV saikosaponins could produce [aglycone−CH_2_OH−OH−H]^−^ and [aglycone−H_2_O−H]^−^ fragment ions through neutral losses at positions C_16_ and C_17_. The above characteristic ions can be used to identify saikosaponins. More notably, the identification process of saikosaponins was systematically summarized, and using this method, 109 saikosaponins were identified or tentatively characterized from the saikosaponins extract of *Bupleurum marginatum var. stenophyllum* (BMS) using UPLC-PDA-Q/TOF-MS with both data-dependent acquisition (DDA) and data-independent acquisition (DIA) modes, of which 25 were new compounds and 60 were first discovered from BMS. Further studies revealed that the saikosaponins profiles of BMS, *Bupleurum chinense* DC (BC), and *Bupleurum marginatum* Wall. ex DC (BMW) were very similar. This work is of great significance for the basic research of the *Bupleurum* species and provides strong technical support to solve the resource problems associated with Radix Bupleuri.

## Introduction

Radix Bupleuri (RB) is a classic herb from the *Bupleurum* species called “chaihu” in Chinese. As one of the most successful and widely used traditional Chinese medicines (TCMs) in Asia, RB is widely used clinically to treat liver disease, fever, chills, tumor, hypochondria, inflammation, and uterine prolapse and has a history of over 2000 years (Ashour and Wink, 2011). According to the Chinese Pharmacopeia, *Bupleurum chinense* DC (BC) and *Bupleurum scorzonerifolium* Willd. are recognized as the official medicinal materials of RB, which are named “North Chaihu” and “South Chaihu,” respectively. With the growing attention to improving health conditions, the development and utilization of RB resources have expanded rapidly, but actual RB resources have gradually decreased due to irrational harvesting and waning fields for forestry. Therefore, it is urgent to search for other *Bupleurum* species that can replace legal RB species as medicine for development and application.

*Bupleurum marginatum var. stenophyllum* (BMS) is a major *Bupleurum* species used in TCM in China and was first recorded in the Southern Yunnan Materia Medica. BMS was introduced from Tibet, due to its high yield, high content of saikosaponins, and low price, and it was widely planted in Gansu and other regions, gradually radiating to Shanxi, Qinghai, and other provinces across China. BMS is an abundant resource and has a long history of medicinal use; related studies have shown that the pharmacological effects of BMS are similar to those of BC (Wang et al., 2019; Wang et al., 2020), and the active ingredients are relatively similar to those of BC (Wang and Liu., 2019), so BMS is considered to be the preferred alternative *Bupleurum* species of BC with good development and application prospects. Thus, it is necessary to conduct detailed basic research on its main active ingredients, the saikosaponins.

**FIGURE 1:**
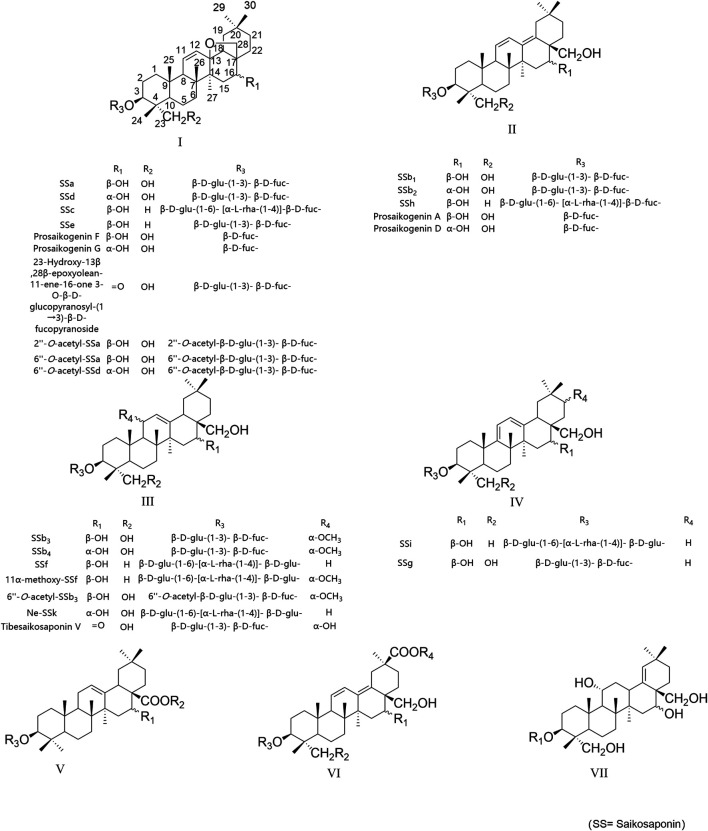
Structures of major saikosaponins.

Saikosaponins are the most important pharmacochemical components of the *Bupleurum* species with multiple pharmacological activities that cover nearly all aspects of the medicinal efficacy of *Bupleurum* medicinal materials (Abe et al., 1982; Zhao et al., 2019; Ren et al., 2019; Wang et al., 2013; Li et al., 2018). There are a large variety of saikosaponins, and to date, over 100 saikosaponins have been isolated from the *Bupleurum* species, and their structures are mainly pentacyclic triterpenoid derivatives, which have mainly been classified into 7 different types based on the aglycone structure: epoxy ether at C_13_, C_28_-position (type I), isocyclic diene (type II), C_12_-ene (type III), homocyclic diene (type IV), C_12_-ene-C_28_-carboxylic acid (type V), isocyclic diene and C_30_-carboxylic acid (type VI), and C_18_-ene (type VII); the main structural formulas are shown in Figure 1. The conjugated sugars are mainly glucose (Glu), rhamnose (Rha), furanose (Fuc), xylose (Xyl), and pentitol. Saccharide chains are usually attached to the aglycone at position C_3_, among which type I saikosaponins only exist in *Bupleurum* plants and consist of the highest content of native saikosaponins in the *Bupleurum* species (Sun et al., 2019; Jiang et al., 2020).

Saikosaponins are not only diverse in structure but also very similar in polarity, and there are many isomers. It is tedious to purify, separate, and identify them using conventional phytochemical separation methods, and the experimental period is long. In recent years, ultra-performance liquid chromatography-mass spectrometry (UPLC-MS) has been widely used in many research fields due to its ability to obtain abundant sample information and efficient component separation, such as rapid identification of chemical components of TCM and drug quality control, speculated by aligning MS information and fragmentation patterns of target compounds with chemical standard substances or literature; the chemical components of TCM can be rapidly and accurately identified (Guo et al., 2016; Qing et al., 2017; Münger et al., 2018; Yang et al., 2019; Ye et al., 2019; Xia et al., 2021). At present, there are still many saikosaponins with structural diversity present at low abundance that remain to be separated and identified by UPLC-MS.

In this study, an efficient sample-processing method was established to extract, purify, and enrich saikosaponins, especially trace saikosaponins at low abundance; we eliminated the interference of non-saikosaponin components and improved their detection efficiency and selectivity. Next, we established an UPLC-PDA-Q/TOF-MS method combined with both DDA and DIA modes to analyze 24 different saikosaponin standard substances with known structures, fully analyzed and summarized their properties such as fragment ions, retention time, and maximum UV wavelength, further applied its summary rules to reliably characterize and identify the total saikosaponin extract from BMS, and compared them with saikosaponins of two other commonly used *Bupleurum* species—BC (the legal RB) and *Bupleurum marginatum* Wall. ex DC (BMW, another major *Bupleurum* species, whose genetic relationship is close to BMS); the objective was to explore the similarities and differences of saikosaponins between BMS and them in order to provide essential data for related research.

## Materials and Methods

### Chemicals and Reagents

Formic acid (LC-MS grade), leucine enkephalin (LC-MS grade), and sodium formate (LC-MS grade) were obtained from Sigma-Aldrich Corporation (MO, United States). Methanol (LC-MS grade), acetonitrile (LC-MS grade), and ammonium acetate (LC-MS grade) were obtained from Fisher Corporation (United States). Ultra-high–purity water was obtained from a Millipore Alpha-Q water purification instrument. The filter membrane (0.22 μm) was obtained from Millipore Corporation (MA, United States).

Saikosaponin a (SSa, batch number 110777-201912) and Saikosaponin d (SSd, batch number 110778-201912) were purchased from China National Institutes for Food and Drug Control. Saikosaponin b_1_ (SSb_1_, batch number 58558-08-0), Saikosaponin b_2_ (SSb_2_, batch number 58316-41-9), Saikosaponin b_3_(SSb_3_, batch number 58316-42-0), Saikosaponin b_4_ (SSb_4_, batch number 58558-09-1), Saikosaponin c (SSc, batch number 20736-08-7), Saikosaponin e (SSe, batch number 64340-44-9), Saikosaponin f (SSf, batch number 62687-63-2), 11α-Methoxysaikosaponin f (11α-methoxyl-SSf, batch number 104109-37-7), Saikosaponin g (SSg, batch number 99365-19-2), Saikosaponin h (SSh, batch number 91990-63-5), Saikosaponin i (SSi, batch number 103629-71-6), Nepasaikosaponin k (Ne-SSk, batch number 405229-61-0), Prosaikogenin d (batch number 103629-72-7), and 23-Hydroxy-13β,28β-epoxyolean-11-ene-16-one 3-O-β-D-glucopyranosyl-(1→3)-β-D-fucopyranoside (batch number 106452-32-8) were purchased from Shanghai Standard Technology Co., Ltd. (Shanghai, China). 6″-*O*- Acetylsaikosaponin-SSb_3_ (6″-*O*-acetyl-SSb_3_, batch number 104109-34-4) and 6″-*O*-Acetylsaikosaponin a (6″-*O*-acetyl-SSa_3_, batch number 64340-46-1) were purchased from Shanghai Yuanye Bio-Technology Co., Ltd. (Shanghai, China). Prosaikogenin a (batch number 99365-21-6), Prosaikogenin f (batch number 99365-20-5), and Prosaikogenin g (batch number 99365-23-8) were purchased from Chengdu Pufei De Biotech Co., Ltd. (Sichuan, China). Tibesaikosaponin V (batch number 2319668-87-5) was purchased from Chengdu Must Biotech Co., Ltd. (Sichuang, China). 6″-O-Acetylsaikosaponin d (6″-*O*-acetyl-SSd, batch number 64340-45-0) and 2″-*O*-Acetylsaikosaponin a (2″-*O*-acetyl-SSa, batch number 102934-42-9) were purchased from Chengdu Weikeqi Biotech Co., Ltd. (Sichuang, China).

The Chinese herbal BMS materials were collected from Gansu Province (China), BC materials were collected from Shanxi Province (China), and BMW materials were collected from Sichuan Province (China). The above samples were confirmed by researcher Xianlong Cheng from China National Institutes for Food and Drug Control.

### Preparation of the Total Saikosaponin Extract From Samples

The samples were pulverized and extracted with 10 times the volume in 70% ethanol (containing 0.05% ammonia water) twice for 4 h under reflux, followed by concentration under reduced pressure. The residue was extracted with petroleum ether, ethyl acetate, and water-saturated n-butanol, respectively; the water-saturated n-butanol part was recovered to extract. We dispersed the above extract by D101 macroporous resin column chromatography, eluting with water, 30% ethanol, 70% ethanol, and 95% ethanol in turn, and finally collected the 70% ethanol fraction to obtain the purified and enriched total saikosaponin extract of samples.

### Preparation of Sample Solutions and Standard Substance Solutions

To obtain 20 mg of the prepared total saikosaponin extract, the materials were accurately weighed and added to a 10-ml brown volumetric flask, and methanol was added on a scale, sealed, and ice-bath sonicated for 5 min, and the solution was filtered using a 0.22-μm microporous filter membrane to obtain the sample solutions. The appropriate amount of each standard substance was weighed accurately and diluted with methanol to the desired concentrations, and the standard substance solutions were obtained.

### UPLC-PDA-Q/TOF-MS Analysis for Saikosaponin Extract and Standard Substance Solutions

We used the ultra-high performance LC System with a Q/TOF-MS, PDA detector, and Masslynx workstation (Waters MS Technologies, Manchester, United Kingdom) to acquire UPLC–high-resolution mass spectrometry (HRMS) raw data. Chromatographic separation was performed on an ACQUITY BEH C_18_ column (150 mm × 2.1 mm, 1.7 μm), maintained at 35°C. The flow rate was 0.3 ml/min, and the injection volume was 2 μL. The mobile phase consisted of (solvent A) 0.05% formic acid in acetonitrile (v/v) and (solvent B) 0.05% formic acid in water (v/v), and the gradient elution was as follows: 0–4 min, 5%–15% A; 4–20 min, 15%–30% A; 20–30 min, 30% A; 30–40 min, 30%–44% A; 40–47 min, 44% A; 47–54 min, 44%–90% A; 54–55 min, 90%–98% A; 55–56 min, 98% A. The detection wavelength range of the PDA detector was set to 200–400 nm.

For MS conditions, the temperature of the electrospray ion (ESI) source was set to 120°C; the cone and capillary voltages were 30 V and 3.0 kV, respectively. High-purity nitrogen was selected as the desolvation gas with the desolvation temperature set to 450°C; high-purity argon was selected as the collision gas. The [M-H]^–^ ion of leucine enkephalin (LE) at m/z 554.2615 was selected as the lock mass in ESI^–^; the [M + H]^+^ ion of LE at m/z 556.2771 was selected as the lock mass in ESI^+^. Sodium formate solution was mass-spectrometrically tuned to ensure the accuracy and reproducibility during the experiment. For the data acquisition mode, the MS^E^ and DDA acquisition modes were selected, and the mass scan range was 100–1,500 Da. In the MS^E^ continuous acquisition mode, the sample data were collected by setting high and low two different collision energy (CE) pathways alternately to obtain molecular ions and fragment ions of target compounds. In terms of the CE, the low CE channel was set to 8 V and the high CE channel was set to 20–70 V. The DDA acquisition mode was set to 5 ion acquisition channels, and other acquisition conditions were the same as the MS^E^ mode.

## Results and Discussion

### Establishment of Sample-Processing Method

Conventional sample-processing methods mostly use methanol to extract saikosaponins for detection, which presents shortcomings. First, numerous non-saikosaponin components present in the extraction solution, which are extracted together with saikosaponins during detection, interfere with the identification of saikosaponins. Second, the extraction efficiency is low, and many trace saikosaponins are not fully extracted, which results in the missed detection. In this study, an efficient sample-processing method was developed to extract, enrich, and purify saikosaponins (especially the trace saikosaponins), eliminate the interference of non-saikosaponin components, and improve their detection efficiency and selectivity.

### Establishment of Detection Method

The range of UV detection wavelengths was set at 200–400 nm based on the structure of the saikosaponins. The data acquisition modes of mass spectrometry included data-dependent acquisition (DDA) and data-independent acquisition (DIA, i.e., MS^E^). The DDA mode reduces the existence of interfering ions because it uses a narrow m/z window to screen target ions. Therefore, it can provide high-quality fragment information, but when valuable ions cannot satisfy the target screening conditions or co-flow with high-intensity ions, these target ions cannot be selected for fragmentation, or the number of parent ions selected will be too large, and this will lead to insufficient acquisition of the total ion chromatograms or base peak ion chromatograms (BPI) of the primary mass spectrum. For the DIA (MS^E^) mode, the primary mass spectrum of ions was obtained at the low CE channel, and fragment ion information was obtained at the CE channel. In theory, the fragment information of all ions can be obtained comprehensively without screening the parent ions in advance. However, if there are many ions flowing concurrently, it is difficult to directly analyze the primary mass spectra and fragment ions, that is, it is difficult to directly determine from which specific parent the fragment ions in the secondary mass spectrum (MS/MS) derive (Law and Lim, 2013; Bateman et al., 2014).

Because saikosaponin compounds present both structural diversity and high structural similarity, even under gradient elution, it is currently difficult to separate all saikosaponin compounds from the baseline completely, in which there are co-efflux components and many trace saikosaponins with a low response. Thus, we combined the DDA and DIA (MS^E^) modes to complement each other to scan and analyze parent ions and product fragment ions of saikosaponins accurately and comprehensively. The ESI^–^ mode provides more abundant structural information of saikosaponin compounds, while the ESI^+^ spectra of saikosaponins provide less information and compounds are subjected to breakage in the low CE scanning channel, which results in more interference with the analysis but can also document the characteristic fragment ions in ESI^+^ and contribute to the determination of the specific position of substituent groups in acetylated/malonylated saikosaponins (Liu et al., 2019). Thus, this approach was used to identify saikosaponins in the ESI^–^, whereas for the identified acetylated/malonylated saikosaponins in ESI^–^, their fragment ions in ESI^+^ were combined to speculate on the specific position of the respective acetyl/malonyl groups.

### UPLC-PDA-Q/TOF-MS Analysis of Saikosaponin Standard Substances

In this study, fragment ions were named according to the nomenclature rules of Domon and Costello (Supplementary Figure S1) (Domon and Costello, 1988). A total of 24 saikosaponin standard substances (classified as types I–IV according to their aglycone structures and their acetylated derivatives were classified separately as one group for analysis) were subjected to analysis and the information obtained regarding the chromatographic, fragmentation behavior and maximum UV wavelength was summarized, their structures are shown in Figure 1, and chromatography, UV absorption, and MS spectrum details for each standard substance are shown in Supplementary Table S1.

#### Analysis of Type I Saikosaponins

**FIGURE 2:**
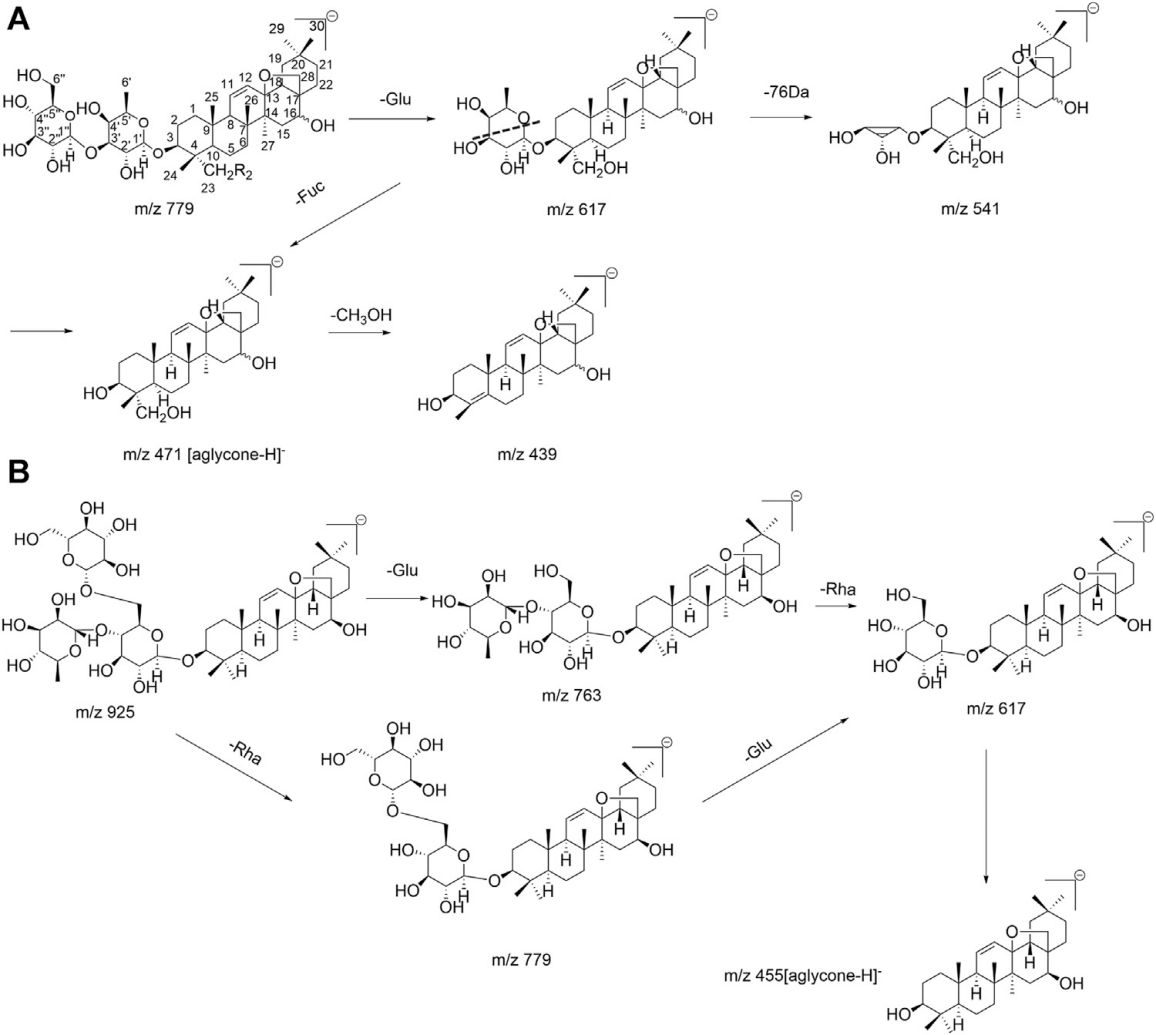
Fragmentation pathway of **(A)** SSa/d and **(B)** SSc.

SSa (β-OH at position C_16_) and SSd (α-OH at position C_16_) were a pair of epimers, with the same molecular ion ([M−H]^−^) at m/z 779; their fragmentation behaviors were similar. In the ESI^–^mode, the fragment ions of the MS/MS spectrum (Supplementary Table S1) were analyzed, and the Y_1_ (m/z 617) ion corresponding to the [M–H]^–^ lost a fragment of 162 Da, indicating that a glucosyl group (Glu, 162 Da, C_6_H_10_O_5_) at the end of the saccharide chain was lost. The X_0_ (m/z 541) ion was produced by the breakage of the Y_1_ (m/z 617) ion, corresponding to the Y_1_ ion with a lost fragment of 76 Da (C_3_H_8_O_2_), which represented the intracyclic breakage of furanose (0, 3-bond breakage). The [Y_1_−76−H]^–^ (m/z 541) fragment ion was produced when the furanose (Fuc, 146 Da, C_6_H_10_O_4_) was directly attached to the aglycone in the structure of saikosaponins; conversely, the presence of the [Y_1_−76−H]^–^ fragment ion was not detected for saikosaponins without furanose. Thus, a fragment of 76 Da (C_3_H_8_O_2_) was lost as the specific fragmentation pathway of furanose, which was consistent with previous reports (Huang et al., 2008). Among the characteristic fragment ions of SSa/SSd was m/z 471 ([M−Glu−Fuc−H]^–^), which corresponded to the molecular ion without the complete saccharide chain and corresponded to the sapogenin ion ([aglycone-H]^−^); the [aglycone−H]^−^ lost a fragment of 32 Da to produce the m/z 439 ([aglycone−CH_3_OH−H]^−^) fragment ion, corresponding to the aglycone with the loss of one molecule of CH_3_OH at position C_4_, which was consistent with the available literature (Zhao et al., 1996). The fragmentation pathway of SSa/SSd is shown in Figure 2A. Similarly, the fragmentation pathways of SSe, Prosaikogenin f, Prosaikogenin g, and other type I saikosaponins are similar to those of SSa/SSd. For SSc, the aglycone structure is different from that of SSa; the group at position C_23_ is methyl (CH_3_), and its fragmentation pathway is shown in Figure 2B.

23-Hydroxy-13β,28β-epoxyolean-11-ene-16-one 3-*O*-β-D-glucopyranosyl-(1→3)-β-D-fucopyranoside presents a carbonyl group (C=O) in the aglycone moiety and its characteristic fragment ions appeared at m/z 407 ([aglycone−CH_3_OH−30−H]^−^) in the ESI^–^mode, corresponding to the [aglycone−H]^−^, and lost a fragment of 30 Da. It was speculated that the C_13_,C_28_-epoxy ether bond was broken first and was followed by a rearrangement reaction of the C=O group at the C_16_ position and breakage. The fragmentation pathway is shown in Supplementary Figure S2.

Retention times of type I saikosaponins showed (Supplementary Table S1) that for a pair of epimers, the retention time of β-OH at position C_16_ was earlier than that of the α-OH epimer (such as SSa/SSd and Prosaikogenin g/Prosaikogenin f). For the type I saikosaponins, their aglycones present only one double bond (no conjugation), so their maximum UV wavelengths were generally around at the terminal ends (190–220 nm); the representative UV spectra are shown in Figure 8A.

#### Analysis of Type II Saikosaponins

**FIGURE 3:**
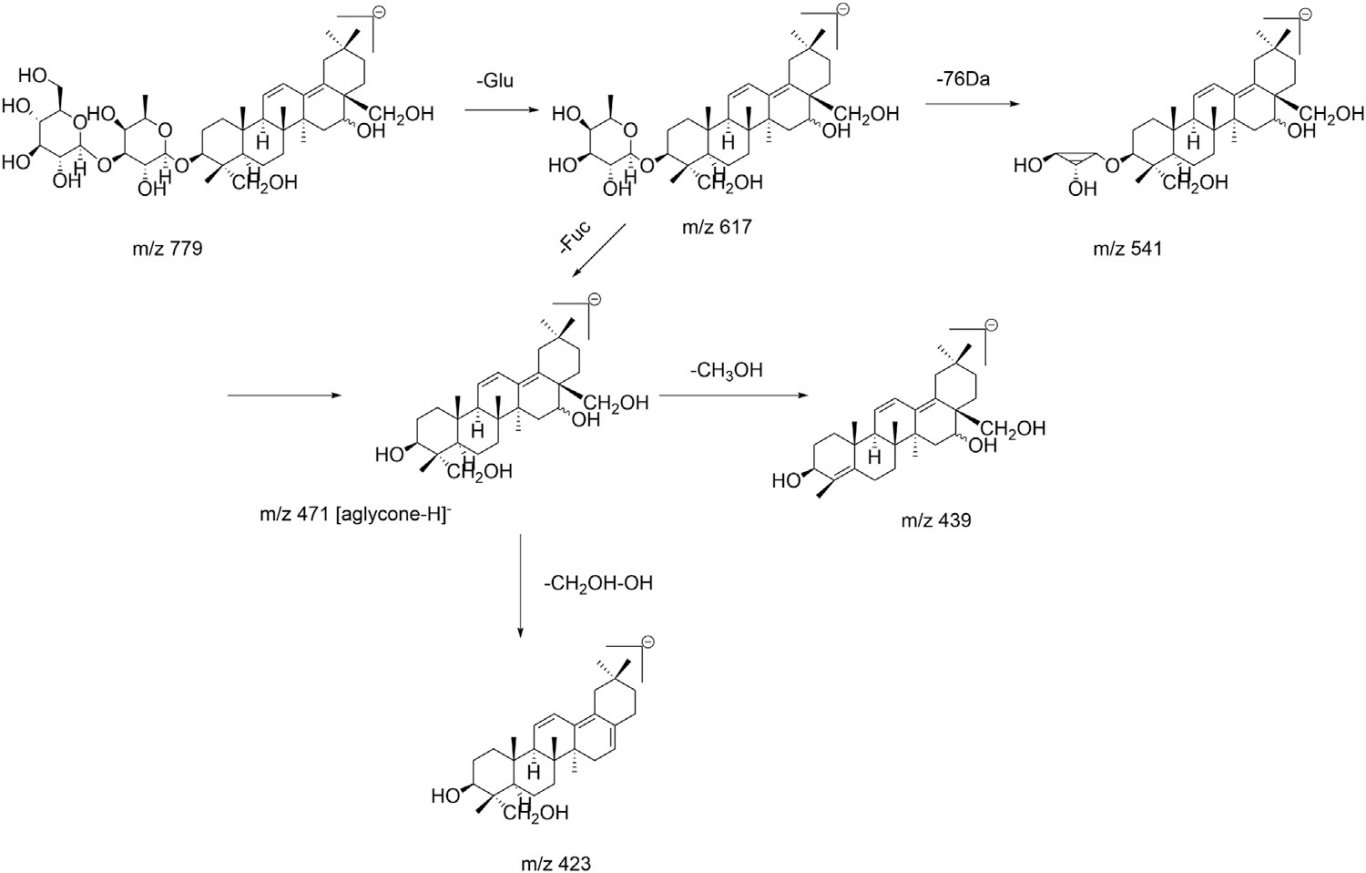
Fragmentation pathway of SSb_1_/SSb_2_.

Epimers SSb_1_ (β-OH at position C_16_) and SSb_2_ (α-OH at position C_16_) are converted from SSa and SSd, respectively. In the ESI^–^mode, the fragmentation behavior of the two epimers was similar to that of SSa/SSd, and they also produced the following fragment ions: m/z 617 ([M−Glu−H]^–^), m/z 541 ([M−Glu−C_3_H_8_O_2_−H]^–^), m/z 471 ([M−Glu−Fuc−H]^–^, corresponding to [aglycone−H]^−^), and m/z 439 ([aglycone−CH_3_OH−H]^−^). Compared with SSa/SSd (type I), SSb_1_/SSb_2_ lost a CH_2_OH group at position C_17_ and a hydroxyl (OH) group at position C_16_ in the aglycone, simultaneously, and generated the m/z 423 ([aglycone−48−H]^−^) fragment ion, which was consistent with the fragmentation behavior found by Liu et al. (Liu et al., 2019). Because SSf presented a single double bond of aglycone and did not produce a m/z 409 ([aglycone−48−H]^−^) fragment ion, it can be inferred that this fragmentation pathway only occurred when two double bonds were conjugated. Interestingly, it was worth noting that this experiment found that the abundance of m/z 423 ([aglycone−CH_2_OH−OH−H]^−^) fragment ion produced by SSb_1_ having the β-OH at the C_16_ position was greater than that of the m/z 439 ([aglycone−CH_3_OH−H]^−^) fragment ion, while the abundance of the m/z 423 fragment ion produced by the SSb_2_ with the α-OH at position C_16_ was lower than that of the m/z 439 fragment ion produced. As shown in Supplementary Figure S3, it is helpful to distinguish between the same pair of epimers in type II saikosaponin. These rules and findings were also applicable to other type II saikosaponins such as Prosaikogenin a, Prosaikogenin d, SSh, etc. The fragmentation pathway of SSb_1_/SSb_2_ is shown in Figure 3. The fragmentation pathway of SSh (the group at the C_23_ position is CH_3_) is shown in Supplementary Figure S4 and also produced a m/z 407 ([aglycone−CH_2_OH−OH−H]^−^) fragment ion. Thus, the [aglycone−48−H]^−^ could be considered diagnostic fragment ions of type II saikosaponins, while the loss of 48 Da (neutral loss of CH_2_OH and OH groups) may also be considered a characteristic loss of type II saikosaponins.

Retention times of type II saikosaponins are reported in Supplementary Table S1. For the pair of epimers, the retention time of β-OH at the C_16_ position was later than that of the α-OH epimer (such as SSb_1_/SSb_2_ and Prosaikogenin a/Prosaikogenin d), which was contrary to that of type I saikosaponins. For the type II saikosaponins, the aglycones presented two double bonds (isocyclic diene conjugation), and the maximum UV wavelengths were generally around 250 nm; the representative UV spectra are shown in Figure 8B.

#### Analysis of Type III Saikosaponins

**FIGURE 4:**
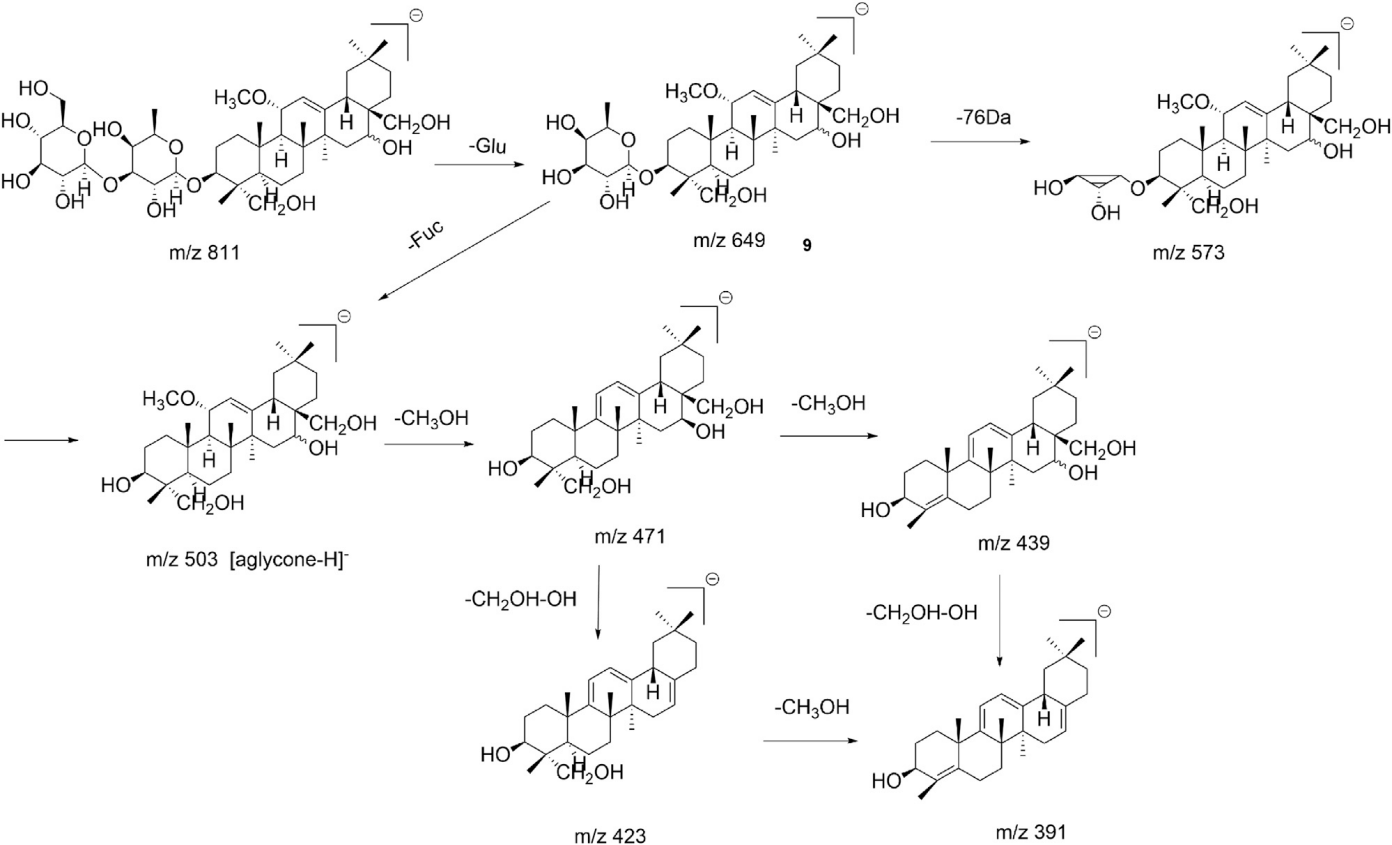
Fragmentation pathway of SSb_3_/SSb_4_.

SSb_3_ (β-OH at position C_16_) and SSb_4_ (α-OH at position C_16_) with the aglycone structure of C_11_ -OCH_3_ are epimers. According to the fragment ions produced in the ESI^–^mode, the aglycone was released at the C_11_ position, one molecule of CH_3_OH (32Da) was lost and produced an aglycone with C_8_-C_11_ double bonds, and the m/z 471 ([aglycone−CH_3_OH−H]^−^) fragment ion was produced. Thus, there were two double bonds (Δ^8,11^ and Δ^12,13^) in the aglycone due to the diene bond conjugation, the CH_2_OH group at position C_17_ and the OH group at position C_16_, which could be lost simultaneously (loss of 48 Da), which produced the m/z 423 ([aglycone−CH_3_OH−CH_2_OH−OH−H]^−^) fragment ion. The loss of C_4_-CH_3_OH produced the fragment ion m/z 391 ([aglycone−2CH_3_OH−CH_2_OH−OH−H]^−^). Based on the m/z 439 ([aglycone −2CH_3_OH−H]^−^), another fragmentation pathway of the aglycone produced the loss of C_11_-CH_3_OH and C_4_-CH_3_OH in turn; the fragmentation pathway of SSb_3_/SSb_4_ is shown in Figure 4. Similarly, for SSf, Ne-SSk, and 11α-methoxy-SSf, the fragmentation pathways were similar and are shown in Supplementary Figures S5–S7. Thus, the fragment ions produced by loss of a 48-Da fragment ([aglycone−CH_2_OH−OH−H]^−^) were also a diagnostic fragment ion of type III saikosaponins in the ESI^−^ mode.

**FIGURE 5:**
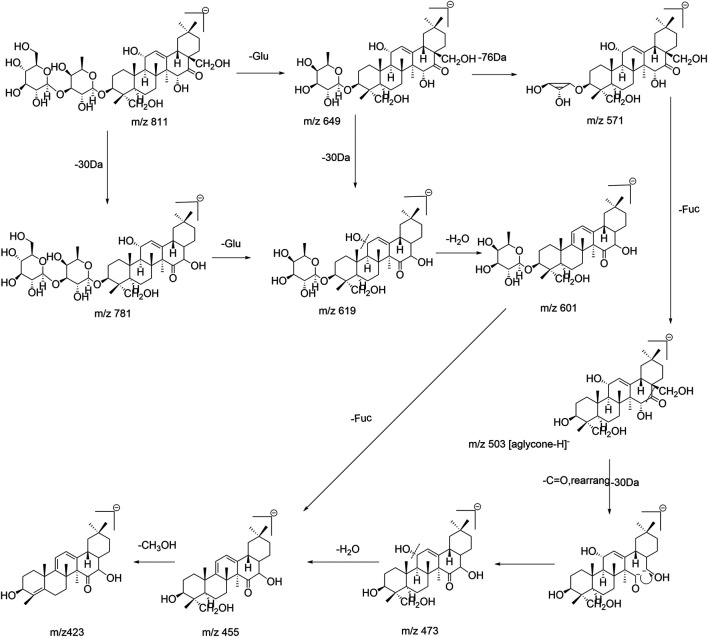
Fragmentation pathway of Tibesaikosaponin V.

The fragmentation behavior of Tibesaikosaponin V with the carbonyl (C=O) group was similar to that of SSb_3_/SSb_4_, but the characteristic fragment ions were produced by the loss of 30 Da (neutral loss of CH_2_O), such as m/z 473 ([aglycone–30–H]^−^). This behavior was the same for 23-hydroxy-13β,28β-epoxyolean-11-ene-16-one 3-*O*-β-D-glucopyranosyl-(1→3)-β-D-fucopyranoside (type I), and all had a carbonyl (C=O) group in the aglycone moiety. The fragmentation pathway is shown in Figure 5. Thus, the characteristic fragment ions produced by loss of 30 Da could be considered the diagnostic fragment ions of saikosaponins containing carbonyl groups in the ESI^−^ mode. This study analyzed and summarized the characteristic fragment ions and fragmentation pathways of saikosaponins containing the C=O group for the first time, which is of great significance and benefit for the identification of these types of saikosaponins.

Retention times of type III saikosaponins revealed (Supplementary Table S1) that in the pair of epimers, the retention time of β-OH at position C_16_ was earlier than that of the α-OH epimer. For type III saikosaponins, similar to type I saikosaponins, the aglycones also had a single double bond (no conjugation), and the respective maximum UV wavelengths were also approximately 190–220 nm. The representative UV spectra are shown in Figure 8C.

#### Analysis of Type IV Saikosaponins

**FIGURE 6:**
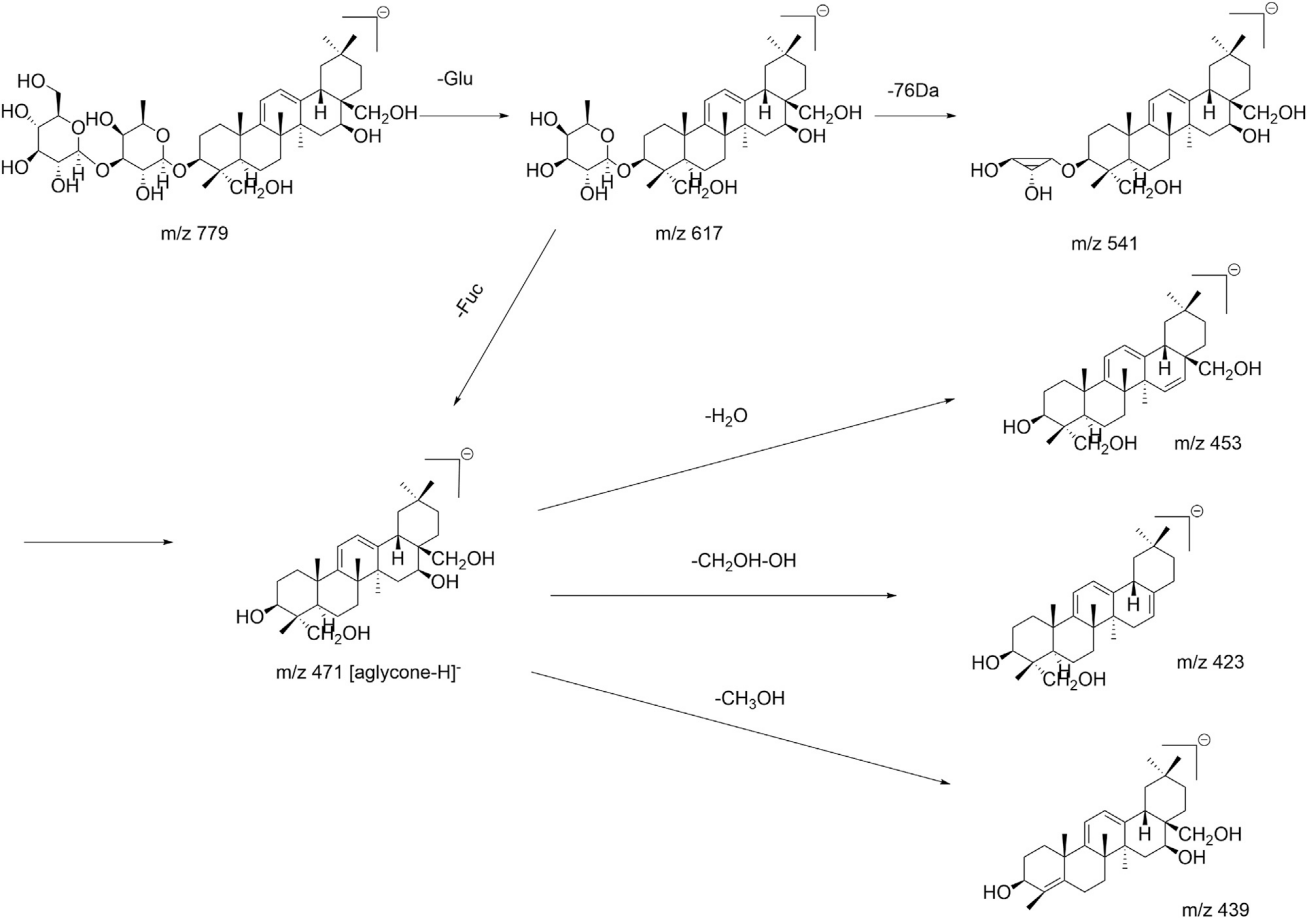
Fragmentation pathway of SSg.

SSg was compared with the type II saikosaponin SSb_1_ (isomer of SSg) generated fragment ions that were essentially the same in ESI^–^, which could produce m/z 423 ([aglycone−CH_2_OH−OH−H]^−^) and other fragment ions of SSb_1_. However, this is the first study to observe the m/z 453 ([aglycone–18–H]^−^) fragment ion from SSg, while the isomer SSb_1_ (type II) or SSa (type I) did not produce the same fragment. It was speculated that due to the homocyclic diene conjugation effect, the OH group can be lost alone; the fragmentation pathway was shown in Figure 6. Thus, in addition to the [aglycone−CH_2_OH−OH−H]^−^, the characteristic fragment ions such as [aglycone−H_2_O−H]^−^ produced by loss of 18 Da could be considered a diagnostic fragment ion of type IV saikosaponins in the ESI^−^ mode.

For type IV saikosaponins, the aglycone moieties present two double bonds (homocyclic diene conjugation), so their maximum UV wavelengths were generally around 282 nm, and the representative UV spectra are shown in Figure 8D.

The maximum UV wavelengths, characteristic fragment ions, and fragmentation pathways of type IV saikosaponins were analyzed and summarized for the first time, and we compared differences in fragmentation patterns with other types of saikosaponins, which is of great significance and contributes to the identification of type IV saikosaponins.

#### Analysis of Acetylated (Malonylated) Saikosaponins

In the ESI^–^ mode, acetylated saikosaponins such as 6″-*O*-acetyl-SSb_3_, 2″-*O*-acetyl-SSa, 6″-*O*-acetyl-SSa, and 6″-*O*-acetyl-SSd generally produced [M–42–H]^−^ and [M–60–H]^−^ fragment ions (Supplementary Table S1), corresponding to the loss of one acetyl (CH_2_CO) group and the loss of one CH_3_COOH group, respectively. Subsequently, a series of fragment ions of the prototype saikosaponins mentioned above were produced. As an example, 6″-*O*-acetyl-SSa fragmentation is shown in Supplementary Figure S8. Prior studies have reported (Huang et al., 2008) that the malonylated saikosaponins produced characteristic [M–H–44]^–^ and [M–H–86]^–^ fragment ions in the ESI^−^ mode. As an example, the fragmentation pathway of 6″-*O*-malonyl-SSa is shown in Supplementary Figure S9.

**FIGURE 7:**
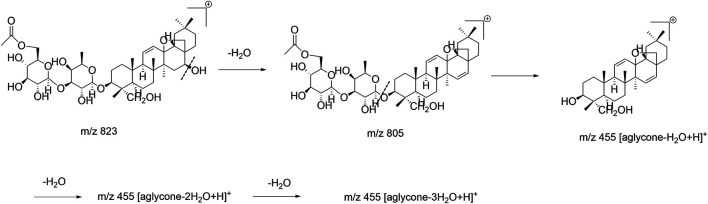
Fragmentation pathway of 6″-*O*-acetyl-SSa in the positive ion mode (ESI^+^).

For acetylated/malonylated saikosaponins in the ESI^–^ mode, the fragment ions of the aglycone-substituted and saccharide-substituted saikosaponins were the same, which made them difficult to distinguish; but in the ESI^+^ mode, their fragment ions differed so that they could easily be distinguished. In the ESI^+^ mode, 6″-*O*-acetyl-SSb_3_, 2″-*O*-acetyl-SSa, 6″-*O*-acetyl-SSa, and 6″-*O*-acetyl-SSd, whose acetyl groups are substituted by the saccharide chain, were analyzed. The fragment ions produced are shown in Supplementary Table S2. The OH group in the aglycone was easily lost as were all produced [aglycone-nH_2_O + H]^+^ (n = 1, 2, or 3) fragment ions. For the 2″-*O*- acetyl-SSa, 6″-*O*-acetyl-SSa, and 6″-*O*-acetyl-SSd, the OH group at position C_16_ was lost first, which produced the m/z 805 ([M–H_2_O + H]^+^) fragment ion, and then OH groups were lost at other positions and produced the fragment ions m/z 455 ([aglycone–H_2_O + H]^+^), m/z 437 ([aglycone–2H_2_O + H]^+^), and m/z 419 ([aglycone–3H_2_O + H]^+^. As a representative example, the fragmentation pathway of 6″-*O*-acetyl-SSa is shown in Figure 7, while that of 6″-*O*-acetyl-SSb_3_ in the ESI^+^ mode is shown in Supplementary Figure S10. These rules and findings were also applicable to malonylated saikosaponins (the malonyl group is substituted by the saccharide chain) according to the related literature (Liu et al., 2019) and also produce [aglycone-nH_2_O + H]^+^ (n = 1 or 2 or 3) in the ESI^+^ mode, while the fragment ions m/z 497 ([aglycone+42–H_2_O + H]^+^) and m/z 479 ([aglycone+42–2H_2_O + H]^+^ indicate that the acetyl group is substituted by the aglycone. For example, the fragmentation pathway in the ESI^+^ mode of 23-*O*-acetyl-SSa (the malonyl group is substituted by the aglycone) (Liu et al., 2019) is shown in Supplementary Figure S11.

Retention times were analyzed according to standard substances and according to that reported in the literature (Zhao et al., 1996; Huang et al., 2008). The retention time of acetylated saikosaponins is later than that of the prototype saikosaponins. The acetyl substituent at different positions of the saccharide chain can affect the retention time; the retention time of the acetyl substituent at the beginning of the saccharide chain is earlier than that of the acetyl substituent at the rear position. For acetylated-SSa, the acetyl group can be substituted at different positions on the terminal glucose of the saccharide chain, so that the retention times for 2″-*O*-acetyl-SSa, 3″-*O*-acetyl-SSa, 4″-*O*-acetyl-SSa, and 6″-*O*-acetyl-SSa range from earlier to later.

Thus, the fragment ions [M–42–H]^−^ and [M–60–H]^−^(or [M–H–44]^–^ and [M–H–86]^–^) are diagnostic fragment ions for acetylated (or malonylated) saikosaponins in the ESI^–^mode, combined with the analysis of their respective m/z values for [aglycone-nH_2_O + H]^+^ in the ESI^+^ mode, and the order of retention times can be combined to speculate the specific position of their acetyl/malonyl group.

#### Summary: Strategy for Identification of Saikosaponins

Among the isomers, several pairs of epimeric saikosaponins such as SSa/SSd, SSb_3_/SSb_4_, SSb_1_/SSb_2_, Prosaikogenin a/Prosaikogenin d, and Prosaikogenin g/Prosaikogenin f differed only in the configuration of the OH group at position C_16_. Of these, saikosaponins with only one double bond in the aglycone moiety (types I and III), and the retention time of β-OH at the C_16_ position was earlier than that of the α-OH epimer, such as SSa/SSd, SSb_3_/SSb_4_, and Prosaikogenin g/Prosaikogenin f. In contrast, for type II saikosaponins (whose aglycone is an isocyclic diene), the retention time of β-OH at position C_16_ was later than that of the α-OH epimer, such as SSb_1_/SSb_2_ and Prosaikogenin a/Prosaikogenin d. For acetylated (or malonylated) saikosaponins, the combination of retention times is helpful to infer the specific position of acetyl (or malonyl) substituents of the saccharide chain.

**FIGURE 8:**
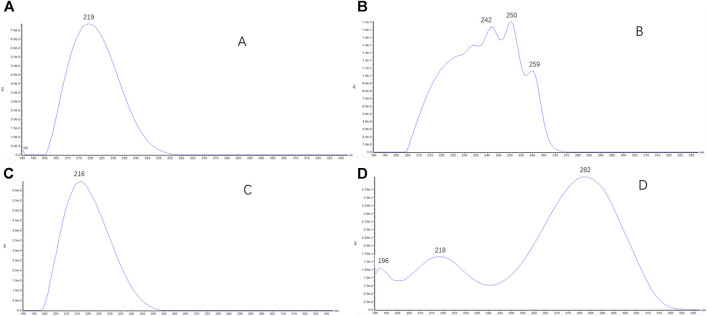
UV spectra of saikosaponins (type I-IV): **(A)** the representative UV spectra (190–220 nm) of type I saikosaponins; **(B)** the representative UV spectra (around 250 nm) of type II saikosaponins; **(C)** the representative UV spectra (190–220 nm) of type III saikosaponins; **(D)** the representative UV spectra (around 282 nm) of type IV saikosaponins.

The maximum UV absorption wavelengths of saikosaponins can be summarized as follows: saikosaponins (such asSSa/SSd/SSc/SSb_3_/SSb_4_/SSe/SSf) with the aglycone as the monoene (types I and III) generally located around the terminal ends, ranged 190–220 nm; saikosaponins (such as SSb_1_/SSb_2_/SSc/SSh) with the aglycone as the isocyclic diene (type II), approximately 250 nm; saikosaponins (such as SSg/SSi) with the aglycone as the homocyclic diene (type IV), approximately 282 nm. The representative UV spectra are shown in Figure 8.

**FIGURE 9:**
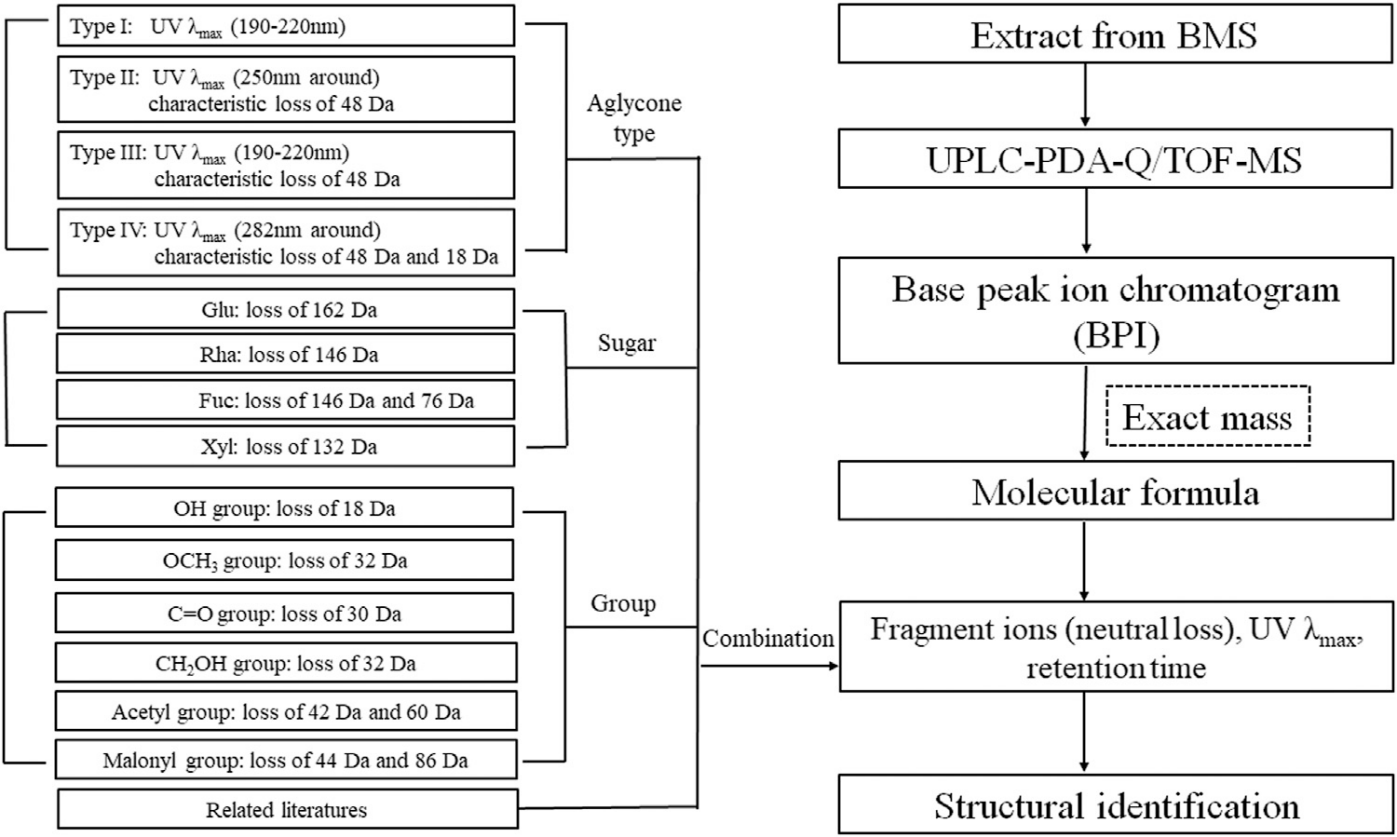
Flowchart for the systematic identification of saikosaponins by UPLC-PDA-Q/TOF-MS combined with a screening method.

The characteristic mass losses and typical fragmentation pathways for different types of saikosaponins can be summarized as follows: all compounds produced a base peak at [M–H]^−^, and the molecular formula of each compound could be determined based on its exact mass. In addition to maximum UV absorption wavelengths, the type of aglycones could be inferred through their characteristic neutral losses of the aglycone moiety, such as type II and III (characteristic loss of 48 Da) and type IV (characteristic loss of 48 and 18 Da). The number and sequences of saccharide chains could be inferred through the characteristic neutral losses of sugar moieties, such as rhamnose (Rha, 146 Da), furanose (Fuc, 146 and 76 Da), xylose (Xyl, 132 Da), and glucose (Glu, 162 Da). Other typical losses were those such as OCH_4_ (32 Da), H_2_O (18 Da), CH_3_OH (32 Da), CH_2_O (30 Da), AcOH/C_2_H_2_O (60 Da/42 Da), and C_3_H_2_O_3_/CO_2_ (80 Da/44 Da), which corresponded to the presence of an OCH_3_ group, an OH group, a CH_2_OH group, a C=O group, an acetyl group, and a malonyl group, respectively. Figure 9 illustrates the strategy for identification of saikosaponins.

### UPLC-PDA-Q/TOF-MS Analysis of Saikosaponin Extracts From BMS

**FIGURE 10:**
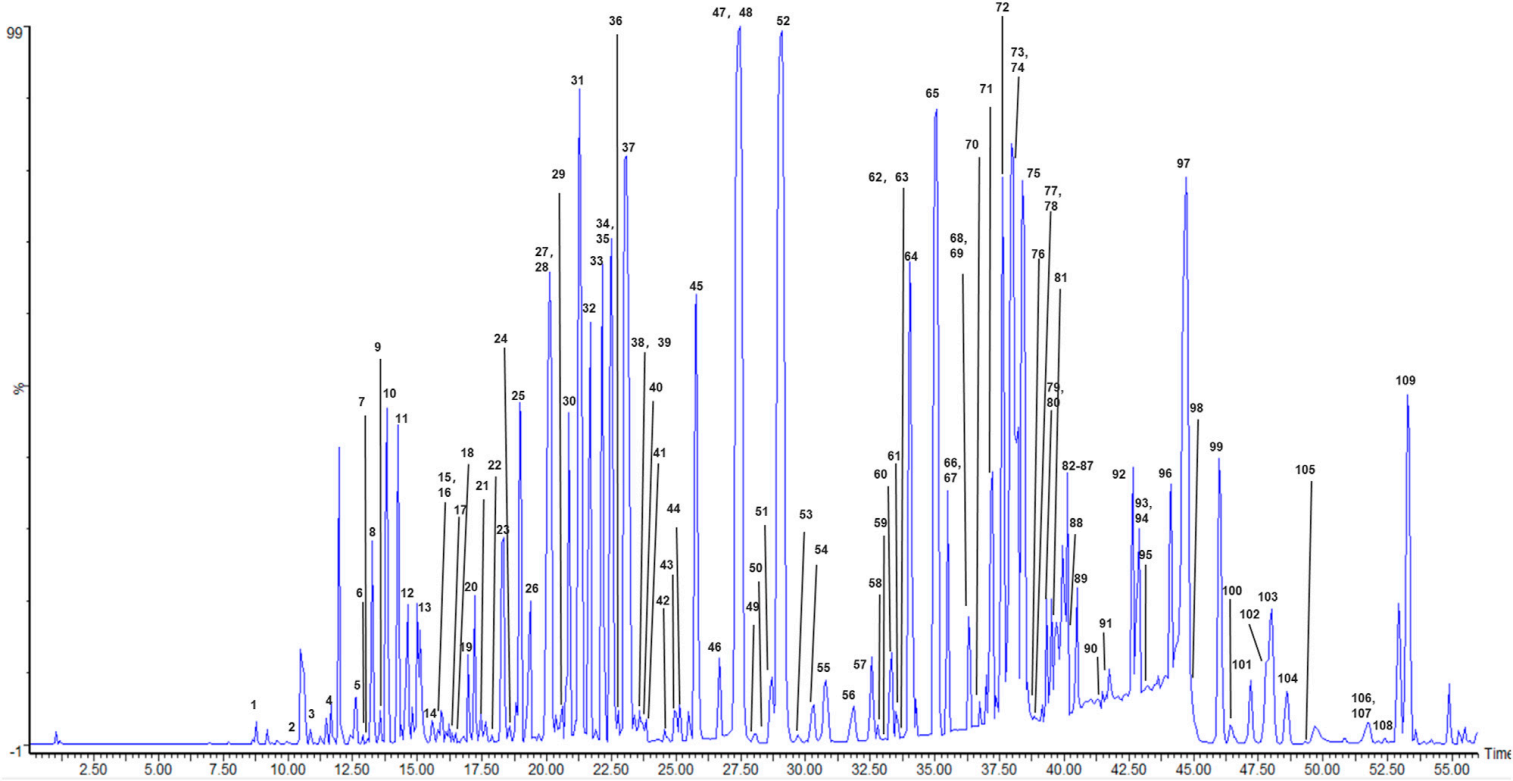
Base peak ion chromatogram of *Bupleurum marginatum var. stenophyllum* (BMS).

In the ESI^–^mode, the base peak ion (BPI) chromatogram of the total saikosaponin extract from BMS is shown in Figure 10. Types I, II, III, and IV and some other types of saikosaponins were identified and initially characterized from the total saikosaponin extract of BMS, and when combined with the respective retention times and fragment ions in the ESI^+^ mode, the specific position of the acetyl/malonyl substituent of acetylated/malonylated saikosaponins could be deduced. A total of 109 saikosaponin compounds were structurally identified and characterized, and the information on their identification, chromatographic retention time (RT), molecular formula, fragment ions, and maximum UV wavelengths is provided in Supplementary Datasheet S1. A total of 22 saikosaponin compounds were confirmed by saikosaponin standards (marked with "*" in Supplementary Datasheet S1), 25 saikosaponins were new compounds (marked with "##" in Supplementary Datasheet S1), and 60 saikosaponins were first discovered in BMS (marked with "##" in Supplementary Datasheet S1).

#### Characterization of Compounds 40, 42, 58, and 71 (Type I)

According to the fragment ions, retention times, and maximum UV wavelength of compounds 40, 42, 58, and 71 (Supplementary Datasheet S1), their characteristic mass losses and UV maximum absorption wavelengths (190–220 nm) are consistent with those of type I saikosaponins, so it was inferred that these compounds had the same aglycones or similar aglycones (with the morehydroxyl substituents at different positions) as type I saikosaponins. 11 type I saikosaponins were identified in this study, including saikosaponin compounds compared with type I related standard substances (acetylated/malonylated type I saikosaponins were described separately below and not included).

In the ESI^−^ mode, the fragment ions of compound 42 had fragment ions m/z 633 ([M− C_6_H_10_O_5_−H]^–^), m/z 557 ([633−C_3_H_8_O_2_−H]^–^, the specific fragmentation pathway of Fuc), and m/z 487 ([633−C_6_H_10_O_4_−H]^–^, aglycone ion), revealing the number and sequence of its saccharide chain, and based on the fragment ions m/z 455 ([aglycone−CH_3_OH−H]^−^) and m/z 437 ([455−H_2_O−H]^−^), which indicated that it comprised one more hydroxyl substituent at the aglycone moiety compared with SSa, compound 42 was identified as clinoposaponin XIV based on a literature review (Miyase and Matsushima, 1997); the fragmentation pathway is shown in Supplementary Figure S12. The same analytical procedure was employed to identify compounds 40, 58, and 71. Compounds 40 and 58 were identified as Sandrosaponin VII (Sánchez-Contreras et al., 2000) and Clinoposaponin XII (Cui et al., 2014). Compound 71 was initially identified as Bupleruoside I or Buddlejasaponin IV (Barrero et al., 2000; Zhu et al., 2018); the two differed only in the order of the saccharide chain and could not be distinguished temporarily. The structures of compounds 40, 58, and 71 are shown in Supplementary Figure S13.

#### Characterization of Compounds 13, 38, 31, 33, 2, 14, 7, 50, 76, 17, 77, and 79 (Type II)

According to the fragment ions, retention times, and maximum UV wavelength (254 nm around), for compounds 13, 38, 31, 33, 2, 14, 7, 50, 76, 17, 77, and 79 (Supplementary Datasheet S1), their characteristic mass losses (characteristic loss of 48 Da) and UV maximum absorption wavelengths (approximately 250 nm) are consistent with those of type II saikosaponins, so they have the same aglycones or similar aglycone structures (probably with the more OH or C=O substituents at different positions) as type II saikosaponins. 15 type II saikosaponins were identified in this study, including saikosaponin compounds compared with related type II standard substances (acetylated/malonylated type II saikosaponins were described separately below and not included).

In the ESI^−^ mode, compounds 31 and 33 shared the same molecular formula. Their fragment ions all showed m/z 779 ([M−C_6_H_10_O_5_−H]^–^), m/z 795 ([M−C_6_H_10_O_4_−H]^–^), m/z 633 ([M−C_6_H_10_O_5_−C_6_H_10_O_4_−H]^–^), and m/z 471 ([M−2C_6_H_10_O_5_−C_6_H_10_O_4_−H]^–^, aglycone ion), which revealed the number and sequences of its saccharide chain. Furthermore, based on the diagnostic fragment ions m/z 439 ([aglycone−CH_3_OH−H]^−^) and m/z 423 ([aglycone−CH_2_OH−OH−H]^−^, produced by the loss of 48 Da (characteristic loss of type II saikosaponins), it was concluded that these compounds had the same aglycone moieties as SSb_1_/SSb_2_. Based on the combined order of their retention times and the abundance of the m/z 423 fragment ion of compound 31, which was smaller than the m/z 439 fragment ion, and compound 33 showing opposite fragments, it was inferred that compound 31 was SSs (α-OH at position C_16_) and compound 33 was SSn (β-OH at position C_16_) (Fang et al., 2017). The fragmentation pathway is shown in Supplementary Figure S14. The same analytical procedure was employed to identify compounds 13 and 38 (having the same molecular formula), and compounds **13** and **38** were identified as SSl (α-OH at position C_16_) and Tibesaikosaponin IV (β-OH at position C_16_), respectively (Yu et al., 2013; Fang et al., 2017); their fragmentation pathways are shown in Supplementary Figure S15.

Employing the same analytical procedure to analyze the characteristic fragment ions and formulas of compounds 2, 14, 7, 50, and 76, their saccharide chains and aglycone structures could be deduced. Through a literature review, compounds 2 and 14 were identified as SSq or its isomer (Huang et al., 2008), and the fragmentation pathway of SSq is shown in Supplementary Figure S16. Compound **7** was identified as SSr (Barrero et al., 2000). The fragment ions and formula of compound 50 were the same as those of SSh, which was identified as Saponin BK1 (isomer of SSh) (Sinha et al., 2021). As for compound 76, its formula and fragment ions were the same as those of SSe (Compound 90, confirmed by the standard substance) and was identified as SSm (Shan et al., 2018). The structures of compounds 7, 50, and 76 are shown in Supplementary Figure S17.

Compounds 17 and 77 shared the same molecular formula and fragment ions, and the diagnostic fragment ions produced from the loss of a 30-Da fragment (the neutral loss of CH_2_O) included m/z 763 ([M–30−H]^−^), m/z 601 ([M–C_6_H_10_O_5_–30−H]^−^), and m/z 455 ([aglycone–30–H]^−^); it was inferred that compounds 17 and 77 had a C=O group on the aglycone moiety. Based on other fragment ions, their saccharide chains and aglycone structures could be deduced, and through a review of the related literature, they were identified as Tibesaikosaponin II or its isomer (Fang et al., 2017); the fragmentation pathway is shown in Supplementary Figure S18. For compound 79, its formula and fragment ions were the same as those of compound 99 (23-hydroxy-13β,28β-epoxyolean-11-ene-16-one 3-O-β-D-glucopyranosyl-(1-3)-β-D-fucopyranoside, confirmed by the standard substance), combined with the maximum UV wavelength (250 nm) and a review of the related literature (Li et al., 2015); it was identified as 3β,23,28-trihydroxyolean-11,13 (18)-diene-16-one 3-*O*-β-D-glucopyranosyl-(1-3)-β-D-fucopyranoside, and its structure is shown in Supplementary Figure S19.

#### Characterization of Compounds 8, 30, 3, 51, 54, 25, and 27 (Type III)

According to the fragment ions, retention times, and maximum UV wavelength of compounds 8, 30, 3, 51, 54, 25, and 27 (Supplementary Datasheet S1), their characteristic mass losses (characteristic loss of 48 Da) and UV maximum absorption wavelengths (190–220 nm) are consistent with those of type III saikosaponins, and it was inferred that these compounds have the same aglycones or similar aglycones as type III saikosaponins. 13 type III saikosaponins were identified in this study, including saikosaponin compounds compared with related type III standard substances (acetylated/malonylated type III saikosaponins were described separately below and not included).

Compounds 8 and 30 shared the same molecular formula as C_42_H_68_O_14_, and the fragment ions of compound 8 produced m/z 633 ([M−C_6_H_10_O_5_−H]^–^), m/z 557 ([633−C_3_H_8_O_2_−H]^–^, the specific fragmentation pathway of Fuc), and m/z 487 ([633−C_6_H_10_O_4_−H]^–^, aglycone ion), which revealed the number and sequences of its saccharide chain, and based on the diagnostic fragment ions m/z 455 ([aglycone−CH_3_OH−H]^−^) and m/z 407 ([455−CH_2_OH−OH−H]^−^), its aglycone moiety could be deduced. Thus, compound 8 was identified as SSt (or Bupleuroside IX) by reviewing the related literature (Yu et al., 2014). The fragmentation pathway of SSt is shown in Supplementary Figure S20. For compound 30, in addition to the above fragment ions of compound 8, additional fragment ions produced from the loss of 30 Da (neutral loss of CH_2_O) appeared, which included fragment ions m/z 765 ([M−30−H]^−^), m/z 603 ([M−C_6_H_10_O_5_−30−H]^−^), and m/z 457 ([aglycone−30−H]^−^), which indicated the presence of a C=O group on the aglycone moiety and was identified as Bupleuroside VI (Liang et al., 2014). The structure is shown in Supplementary Figure S21.

Compound 3, based on the fragment ions and formula, was identified as Rotundioside P by reviewing the related literature (Fujioka et al., 2006), and its fragmentation pathway is shown in Supplementary Figure S22. The same analytical procedure was employed to identify compounds 25 and 27, which presented an additional hydroxyl substituent at the aglycone moiety compared with compounds SSa/SSd, and combined with the order of the respective retention times, it was speculated that compound 25 was hydroxysaikosaponin a and compound 27 was hydroxysaikosaponin d (Huang et al., 2008); both structures are shown in Supplementary Figure S23. Compounds 51 and 54 shared the same molecular formula and fragment ion pattern as SSf (compound 52, confirmed by the standard substance) and were identified as the isomer of SSf, in which we speculated that the position of the double bond was different from that of SSf.

#### Characterization of Compounds 6, 9, and 10 (Types IV and VI)

For compound 6, the fragment ions m/z 647 ([M−C_6_H_10_O_5_−H]^–^), m/z 571 ([647−C_3_H_8_O_2_−H]^–^, the specific fragmentation pathway of Fuc), and m/z 501 ([647−C_6_H_10_O_4_−H]^–^, aglycone ion) revealed the number and sequences of the saccharide chain. According to the diagnostic fragment ions m/z 453 ([aglycone−CH_2_OH−OH−H]^−^) and m/z 483 ([aglycone−H_2_O−H]^−^) and combined with the maximum UV wavelength (282 nm), it was inferred that compound 6 had a similar aglycone moiety to type IV saikosaponins and was speculated to be an isomer of Bupleuroside V, with the following structure, 3β, 16α, 23,28-Tetrahydroxy-olean-9,12 (13)-dien-29-oic acid 3-*O*-β- D-glucopyranosyl-(1-3)-β-D-fucopyranoside. Its fragmentation pathway is shown in Supplementary Figure S24. 3 type IV saikosaponins were identified in this study, including saikosaponin compounds compared with related type IV standard substances.

For compound 10, the number and sequence of its saccharide chain was deduced from the fragment ions (Supplementary Datasheet S1) m/z 453 ([aglycone−CH_2_OH−OH−H]^−^) and m/z 469 ([aglycone−CH_3_OH−H]^−^), but without m/z 483 ([aglycone−H_2_O−H]^−^), and combined with its maximum UV wavelength (251 nm) and formula, it was identified as Bupleuroside V using a literature review (Yoshikawa, 1997). Its fragmentation pathway is shown in Supplementary Figure S25. The same analytical procedure was employed to identify compound 9, which was identified as 3β,16α,23,28-tetrahydroxy-olean-11,13 (18)-dien-30-oicacid-3-*O*-β-D-glucopyranosyl-(1-2)-β-D-glucopyranosyl-(1-3)-β-D-fucopyranoside (Liang et al., 2013); its structure is shown in Supplementary Figure S26. Compounds 9 and 10 belonged to the type VI saikosaponins.

#### Characterization of Compounds 1, 5, 11–12, 15–16, 19–23, 26, 29, 32, 35, 41, 44–45, 49, 60, 68, 72, 74, and 94–96 (Other Types)

According to the formula, fragment ions, retention times, and maximum UV wavelength of compounds 1, 5, 11–12, 15-16, 19–23, 26, 29, 32, 35, 41, 44–45, 49, 60, 68, 72, 74, and 94–96 (Supplementary Datasheet S1), the number and sequences of the saccharide chain can be deduced. In addition, the structures of the aglycone can be identified through further analysis of their fragment ions.

For compound 1, its molecular ion and fragment ions were 18 Da more than those of compound 8 (SSt), and combined with its formula**,** this suggested it had an additional hydroxyl substituent at the aglycone moiety compared with SSt; thus, compound 1 was tentatively identified as hydroxysaikosaponin t. Its fragmentation pathway is shown in Supplementary Figure S27. The same analytical procedure was employed to identify compounds 23, 11, 35, 44, 60, and 49. Compound 23 had an additional hydroxyl substituent at the aglycone moiety compared with SSh (compound 56, confirmed by the standard substance); thus, compound 23 was tentatively identified as hydroxysaikosaponin h (11α-hydroxy-SSh). Similarly, compounds 11, 35, 44, and 60 had an additional hydroxyl substituent at the aglycone moiety compared with SSc (compound 47, confirmed by the standard substance); thus, they were tentatively identified as hydroxysaikosaponin c (11α-hydroxy-SSc) or its isomer (Ebata et al., 1996); for compound 49, its retention time followed that of hydroxysaikosaponin a and hydroxysaikosaponin d (compounds 25 and 27). The diagnostic fragment ions m/z 439 ([aglycone−CH_3_OH−H]^−^) and m/z 423 ([aglycone−CH_2_OH−OH−H]^−^) and the abundance of the fragment ion m/z 423 were smaller than the fragment m/z 439; thus, it was speculated to be hydroxysaikosaponin b_2_ (α-OH at position C_16_). The structures of hydroxysaikosaponin c, hydroxysaikosaponin h, and hydroxysaikosaponin b_2_ are shown in Supplementary Figure S28.

For compound 32, its formula and fragment ions indicated that it had an additional OC_2_H_5_ substituent at the aglycone moiety compared with SSs/SSn (compound 31/33), based on the abundance of the m/z 423 ([aglycone−OC_2_H_5_−48−H]^−^) fragment ion, which was smaller than m/z 439 ([aglycone−OC_2_H_5_−32−H]^−^) when, and combined with its retention time, it was identified as 11α-ethoxyl-SSs (α-OH at position C_16_). Similarly, compounds 72 and 74 were identified as 11α-ethoxyl-SSb_2_ and 11α-ethoxyl-SSb_1_, respectively, while compounds 96 and 45 were identified as 11α-butoxyl-SSb_2_ and 11α-ethoxyl-SSh, respectively. Their fragmentation pathways are shown in Supplementary Figure S29–S32.

Compounds 4, 12, 16, 19, and 20 shared the same molecular formula and fragment ions, and they could be deduced to have one less double bond than SSq (compounds 2 and 14), so they were identified as dihydro-SSq or its isomer (such as 11,12-dihydro-SSq, 13,18-dihydro-SSq, and other possible isomers); their possible structures are shown in Supplementary Figure S33.

Using the same approach, compound 41 was deduced to have one less double bond than Tibesaikosaponin I (compound 29), and combined with the maximum UV wavelength (254 nm), it was tentatively identified as Δ^21,22^-Tibesaikosaponin I; the structure is shown in Supplementary Figure S34. For compounds 29 and 94, it was speculated that their fragmentation pathways were similar to that of Tibesaikosaponin II, but there were no fragment ions produced by the loss of 30 Da, and thus, they were identified as Tibesaikosaponin I or its isomer (Fang et al., 2017), and presumably the C=O group was conjugated with the adjacent olefinic bond, which stabilized the C=O group and was not easy to lose; its fragmentation pathway is shown in Supplementary Figure S35.

Based on the analysis of the formulas, fragment ions, retention times, and maximum UV wavelengths, combined with a review of the literature, compound 21 was identified as Magnoside B (Haddad et al., 2012), and its fragmentation pathway is shown in Supplementary Figure S36. Compound 22 was identified as 3-*O*-[α-L-Rhamnopyranosyl (1–4)-β-D-glucopyranosyl] oleanolic acid 28-*O*-β-D-glucopyranosyl ester (Achouri et al., 2017). Compounds 5 and 15 were identified as (3β,21β,22α)-28-[[2-*O*-(6-Deoxy-α-L-mannopyranosyl)-β-D-glucopyranosyl]oxy]-21,22-dihydroxyolean-12-en-3-yl 6-*O*-β-D-glucopyranosyl-β-D-glucopyranoside or its isomers (Xiao et al., 2013). Compound 68 was identified as (3β,4α,16α)-3,16,23-trihydroxyoleanan-28-yl *O*-6-deoxy-α-L-mannopyranosyl-(1–4)-*O*-[β-D-glucopyranosyl-(1–6)]-β-D-glucopyranoside (Luo and Jin, 1991). Compound 95 was identified as Bupleuroside XI (Yoshikawa, 1997). All structures are shown in Supplementary Figure S37.

#### Characterization of Compounds 18, 36, 39, 43, 46, 53, 55, 57, 59, 61–63, 66, 67, 69, 78, 80–83, 85, 87–88, 91–93, 98, 100–104, and 106–109 (Acetylated/Malonylated Saikosaponins)

The mass spectra fragmentation information of the acetylated/malonylated saikosaponins in the ESI^+^ mode is shown in Supplementary Table S3. 35 acetylated/malonylated saikosaponins were identified in this study, including saikosaponin compounds compared with related standard substances.

Compounds 18, 53, 55, 57, 59, 61–63, 66–67, 69,78, 80–83, 85, 87–88, 92–93, 98, 100, 102–104, and 109 all produced [M–42–H]^−^ and [M–60–H]^−^ fragment ions as diagnostic ions (Supplementary Datasheet S1); thus, they were identified as acetylated saikosaponins.

The compounds 81, 87, 88, 92, 55, 62, 66, 102, 103, 104, 109, 78, 83, 93, and 98 were analyzed, and according to Supplementary Table S3, all fragment ions in the ESI^+^ mode were m/z 455 ([aglycone−H_2_O + H]^+^), m/z 437 ([aglycone−2H_2_O + H]^+^), and m/z 419 ([aglycone−3H_2_O + H]^+^), indicating that their acetyl groups were all substituted by the saccharide chain. In the ESI^−^ mode, in addition to m/z 779 ([M–42–H]^−^) and m/z 761 ([M–60–H]^−^), the remaining fragment ions of compounds 81, 87, 88, and 92 were the same as those of SSa/SSd, and their retention times followed those of SSa but were before SSd; thus, they were identified as acetylated derivatives of SSa. For SSa, SSd, SSb_1_, and SSb_2_, the OH group at the 1″ position of the terminal glucose of the saccharide chain was attached to rhamnose, and only four OH groups at positions 2″, 3″, 4″, and 6″ were substituted by an acetyl group. Based on the order of their respective retention times, compounds 81, 87, 88, and 92 were identified as 2″-*O*-acetyl-SSa, 3″-*O*-acetyl-SSa, 4″-*O*-acetyl-SSa, and 6″-*O*-acetyl-SSa, respectively. Of these, compounds 81 and 92 were confirmed by the standard substance. Similarly, the retention times of compounds 102, 103, 104, and 109 followed SSd, and compound 109 was 6″-*O*-acetyl-SSd by comparison with the standard substance; according to the order of retention times, the compounds 102, 103, and 104 were inferred to be 2″-*O*- acetyl-SSd, 3″-*O*-acetyl-SSd, and 4″-*O*-acetyl-SSd, respectively. For compounds 78, 83, 93, 98, and 100, the abundance of the m/z 423 fragment ion was smaller than that of the m/z 439 fragment ion, indicating that they were acetylated derivatives of SSb_2_. Combined with the order of their retention times, compounds 78, 83, 98, and 100 were 2″-*O*-acetyl-SSb_2_, 3″-*O*-acetyl-SSb_2_, 4″-*O*-acetyl-SSb_2_, and 6″-*O*-acetyl-SSb_2_, respectively. The abundance of the m/z 423 fragment ion of compound 93 was slightly higher than that of m/z 439, which indicated that it was an acetylated derivative of SSb_1_ (*O*-acetyl-SSb_1_). The structures of these acetylated saikosaponins are shown in Supplementary Figure S38.

For compounds 53, 55, 62, and 66 in the ESI^–^mode, in addition to fragment ions such as m/z 925 ([M−42−H]^–^) and m/z 907 ([M−60−H]^–^), the other fragment ions were the same as those of SSc; thus, these were acetylated derivatives of SSc. Their fragment ions in the ESI^+^ mode are shown in Supplementary Table S3 and in combination with the fragment ion in the ESI^–^mode m/z 779 ([M−42−C_6_H_10_O_4_−H]^–^) and m/z 761 ([M−60−C_6_H_10_O_4_−H]^–^), it was deduced that the acetyl group was substituted by the OH group of terminal glucose of the saccharide chain, rather than that of the aglycone. For SSc, the OH group at the 1‴ position of the terminal glucose of the saccharide chain was attached to rhamnose, and only four OH groups at positions 2‴, 3‴, 4‴, and 6'" could be substituted by an acetyl group. Based on the order of their retention times, the 4 compounds were identified as 2‴-*O*-acetyl-SSc, 3‴-*O*-acetyl-SSc, 4‴-*O*-acetyl-SSc, and 6‴-*O*-acetyl-SSc, respectively. The fragmentation pathway in the ESI^–^mode for 6‴-*O*-acetyl-SSc is shown as a representative example in Supplementary Figure S39. Similarly, compounds 57, 59, 63, and 69 were identified as 2‴-*O*-acetyl-SSf, 3‴-*O*-acetyl-SSf, 4‴-*O*-acetyl-SSf, and 6‴-*O*- acetyl-SSf, respectively. Their structures are shown in Supplementary Figure S40.

Based on the fragment ions, compounds 67, 70, 80, and 85 were acetylated derivatives of SSb_3_/SSb_4_, and according to their fragment ions in the ESI^+^ mode (Supplementary Table S3), their acetyl groups were substituted by saccharide chains. Of these, compound 67 had the shortest retention time, which was 2″-*O*-acetyl-SSb_3_. After comparison with the standard substance, compound 80 was deduced to be 6″-*O*-acetyl-SSb_3_, while compound 85 had a retention time that followed that of compound 80 and was thus deduced to be 6″-*O*-acetyl-SSb_4_; both structures are shown in Supplementary Figure S41.

The same analytical procedure was employed to identify compounds 36, 39, and 46 in the ESI^–^mode, and the abundance of the m/z 423 ([aglycone−48−H]^−^) fragment ion was smaller than that of the fragment ion m/z 439 ([aglycone −32−H]^−^) for all compounds; thus, they were identified as acetylated derivatives of SSs (α-OH at position C_16_). Combined with their fragment ions in the ESI^+^ mode (Supplementary Figure S28), the acetyl groups were all substituted by the terminal glucose of the saccharide chain. According to the order of the respective retention times, compounds 36, 39, and 46 were identified as three of 2‴-*O*-acetyl-SSs, 3‴-*O*-acetyl-SSs, 4‴-*O*-acetyl-SSs, and 6‴-*O*-acetyl-SSs, and the respective structures are shown in Supplementary Figure S42. With 6‴-*O*-acetyl-SSs as a representative example, the fragmentation pathway in ESI^–^is shown in Supplementary Figure S43. Similarly, compound 18 was identified as *O*-acetyl-Bupleuroside V. For compound 61, based on its formula and fragment ions in the ESI^–^mode, it was speculated that the C_11_ position had an additional OCH_3_ substituent at the aglycone compared with *O*-acetyl-SSs, according to its fragment ion pattern in ESI^+^ (Supplementary Table S3 **)**, which indicated that an acetyl group was substituted by the aglycone; it was identified as 23-*O*-acetyl-(11α-methoxyl-SSs), and its fragmentation pathway in the ESI^–^mode is shown in Supplementary Figure S44.

Compounds 106 and 108 were analyzed in ESI^–^, their fragment ions all appeared to be m/z 821 ([M−42−H]^–^), m/z 779 ([M−84−H]^–^), and m/z 761 ([M−60−42−H]^–^), and the remaining fragment ions were the same as those of SSa/SSd, which indicated that they were diacetyl derivatization products of SSa/SSd. Supplementary Table S3 indicated that their acetyl groups were all substituted by the saccharide chain; according to their retention times, compounds 106 and 108 all followed the SSd; thus, they were identified as *O*-diacetyl-SSd.

For compounds 82 and 101 in the ESI^–^mode, m/z 821 ([M−44−H]^–^) and m/z 779 ([M−86−H]^–^) fragment ions were produced, and the remaining fragment ions were the same as those of SSa/SSd, which indicated that they were malonylated derivatives of SSa/SSd. As shown in Supplementary Table S3, the malonyl groups were all substituted by the saccharide chain, and after assessing their retention time, compound 82 followed SSa and preceded SSd, and compound 101 followed SSd; thus, compound 82 was *O*-malonyl-SSa and compound 101 was *O*-malonyl-SSd. Similarly, compound 43 was identified as *O*-malony-hydroxysaikosaponin c.

Compounds 91 and 107 were analyzed in the ESI^–^ mode, their fragment ions all appeared at m/z 863 ([M−44−H]^–^), m/z 821 ([M−86−H]^–^), and m/z 779 ([M−42−86−H]^–^), and the remaining fragment ions were the same as those of SSa/SSd, which indicated that their structures contained an additional malonyl substituent and an additional acetyl substituent compared with SSa/SSd. Supplementary Table S3 indicated that their malonyl and acetyl groups were substituted by the saccharide chain. Analyzing their retention times, compound 91 followed SSa and preceded SSd, and compound 107 followed SSd; thus, the former was *O*-malonyl-acetyl-SSa, and the latter was *O*-malonyl-acetyl-SSd.

### UPLC-PDA-Q/TOF-MS Analysis of the Saikosaponin Extract of BC and BMW

The same sample processing and UPLC-PDA-Q/TOF-MS detection methods were applied to identify saikosaponins of BC and BMW, which were then compared with BMS, to explore the similarities and differences of saikosaponins from three *Bupleurum* species. As shown in Supplementary Figure S45, except for a few individual saikosaponins (compounds 15 and 96 were not found in BC, compounds 15, 83 and 96 were not found in BMW), the saikosaponins in BMS were almost the same as those in BC and BMW, which have a material basis as an alternative variety of BC and BMW.

## Discussion

In this study, we used UPLC-PDA-Q/TOF-MS technology to identify saikosaponins from *Bupleurum* species. In previous studies on identification of saikosaponins by UPLC-MS, most of the sample-processing methods used organic reagents such as methanol to crudely extract the saikosaponins from samples, and the saikosaponin components were not further enriched and purified, which may cause omission and interference in the detection of saikosaponin components. In most studies, only a small amount of saikosaponin standards (0–12 standards) were used for analysis; the fragmentation pathways, characteristic loss, retention times, and UV spectral features of saikosaponin standards were not thoroughly analyzed and comprehensively summarized, so not only could some saikosaponins and their isomers not be accurately distinguished and structurally identified but also the types and numbers of saikosaponins identified in *Bupleurum* species were not many, usually dozens of them, and new saikosaponins are rarely found. Compared with previous studies on the identification of saikosaponins by UPLC-MS, the significance and advantages of our established method are summarized below. First, an effective sample-processing method was established, which not only purifies and enriches the saikosaponins but can also eliminate the interference of non-saikosaponins for subsequent analysis. Second, we combined DDA and DIA modes to complement each other and more accurately and comprehensively scanned and analyzed parent ions and product fragment ions of saikosaponins. Third, our study was the most comprehensive characterization of saikosaponins in *Bupleurum* species to date. In this study, the mass spectrometric fragmentation rules, UV spectral features, and chromatographic behaviors of different types of saikosaponins were first investigated and fully summarized using as many as 24 saikosaponin standard substances, the reliability of isomer structures was greatly improved, and a total of 109 saikosaponins were identified, of which 25 were new compounds. Fourth, this paper analyzed and summarized the characteristic fragment ions and fragmentation pathways of saikosaponins containing carbonyl groups and type IV saikosaponins for the first time, based on which these types of saikosaponins were identified, which is of great significance to the expansion of the identification of saikosaponin types.

## Conclusion

Saikosaponins are the most important and prominent medicinal components in *Bupleurum* medicinal materials; thus, it is important to identify and analyze saikosaponins in BMS and other *Bupleurum* species. In this study, the fragmentation pathways of different types of saikosaponins were investigated by UPLC-PDA-Q/TOF-MS. Based on accurate exact mass and the elemental compositions of the fragment ions of 24 standard substances, the mass spectrometric fragmentation pathways, UV spectral features, and chromatographic behaviors were proposed and applied to identify the saikosaponins in extracts from BMS. A total of 109 saikosaponins were identified and characterized, of which 25 were new compounds and 60 were first discovered in BMS. Further studies revealed that the saikosaponins in BMS were almost the same as those in BC and BMW, which indicates that there is a rationale for BMS to be used as an alternative to BC and BMW as medicine. This study provided a foundation for further quality control, pharmacological and pharmacodynamic studies, and the development and application of saikosaponins extracted from BMS and present in other *Bupleurum* species. In the future, we will also conduct detailed study regarding the remaining components of BMS, such as flavonoids, volatile oils, and polysaccharides.

## Data Availability

The original contributions presented in the study are included in the article/Supplementary Material; further inquiries can be directed to the corresponding authors.

## References

[B1] Abe H. Sakaguchi M. Odashima S. Arichi S. (1982). Protective Effect of Saikosaponin-D Isolated from Bupleurum Falcatum L. On CCl4-Induced Liver Injury in the Rat. Naunyn-Schmiedeberg's Arch. Pharmacol. 320 (3), 266–271. 10.1007/BF00510139 7133157

[B2] Achouri A. Derbré S. Medjroubi K. Laouer H. Séraphin D. Akkal S. (2017). Two New Triterpenoid Saponins from the Leaves of Bupleurum Lancifolium (Apiaceae). Nat. Product. Res. 31 (19), 2286–2293. 10.1080/14786419.2017.1324960 28475369

[B3] Ashour M. L. Wink M. (2011). Genus Bupleurum: a Review of its Phytochemistry, Pharmacology and Modes of Action. J. Pharm. Pharmacol. 63 (3), 305–321. 10.1111/j.2042-7158.2010.01170.x 21749378 PMC7197585

[B4] Barrero A. F. Haïdour A. Sedqui A. Mansour A. I. Rodríguez-García I. López A. (2000). Saikosaponins from Roots of Bupleurum Gibraltaricum and Bupleurum Spinosum. Phytochemistry 54 (8), 741–745. 10.1016/s0031-9422(00)00177-1 11014258

[B5] Bateman N. W. Goulding S. P. Shulman N. J. Gadok A. K. Szumlinski K. K. MacCoss M. J. (2014). Maximizing Peptide Identification Events in Proteomic Workflows Using Data-dependent Acquisition (DDA). Mol. Cell Proteom. 13 (1), 329–338. 10.1074/mcp.M112.026500 PMC3879624 23820513

[B6] Cui B.-S. Qiao Y.-Q. Yuan Y. Tang L. Chen H. Li Y. (2014). Hepatoprotective Saikosaponin Homologs from Comastoma Pedunculatum. Planta Med. 80 (17), 1647–1656. 10.1055/s-0034-1383123 25251563

[B7] Domon B. Costello C. E. (1988). A Systematic Nomenclature for Carbohydrate Fragmentations in FAB-MS/MS Spectra of Glycoconjugates. Glycoconj. J. 5, 397–409. 10.1007/BF01049915

[B8] Ebata N. Nakajima K. Hayashi K. Okada M. Maruno M. (1996). Saponins from the Root of Bupleurum Falcatum. Phytochemistry 41 (3), 895–901. 10.1016/0031-9422(95)00720-2 8835463

[B9] Fang W. Yang Y.-J. Guo B.-L. Cen S. (2017). Anti-influenza Triterpenoid Saponins (Saikosaponins) from the Roots of Bupleurum Marginatum Var. Stenophyllum. Bioorg. Med. Chem. Lett. 27 (8), 1654–1659. 10.1016/j.bmcl.2017.03.015 28314599

[B10] Fujioka T. Yoshida K. Shibao H. Nagao T. Yoshida M. Matsunaga K. (2006). Antiproliferative Constituents from Umbelliferae Plants. IX. New Triterpenoid Glycosides from the Fruits of Bupleurum Rotundifolium. Chem. Pharm. Bull. 54 (12), 1694–1704. 10.1248/cpb.54.1694 17139105

[B11] Guo M. N. Liu S. X. Zhao Y. M. Zhang T. J. Liu D. L. (2016). Analysis on Chemical Constituents in Bupleuri Radix by HPLC-Q-TOF-MS. Chin. Tradit. Herbal Drugs 47 (12), 2044–2052. 10.7501/j.issn.0253-2670.2016.12.005

[B12] Haddad M. Lelamer A. Moreno Y Banuls L. Carraz M. Vasquez P. Vaisberg A. (2012). *In Vitro* growth Inhibitory Effects of 13, 28-epoxyoleanane Triterpene Saponins in Cancer Cells. Planta Med. 78 (11), PI179. 10.1055/s-0032-1320867

[B13] Huang H.-Q. Zhang X. Lin M. Shen Y.-H. Yan S.-K. Zhang W.-D. (2008). Characterization and Identification of Saikosaponins in Crude Extracts from threeBupleurumspecies Using LC-ESI-MS. J. Sep. Sci. 31 (18), 3190–3201. 10.1002/jssc.200800120 18763253

[B14] Jiang H. Yang L. Hou A. Zhang J. Wang S. Man W. (2020). Botany, Traditional Uses, Phytochemistry, Analytical Methods, Processing, Pharmacology and Pharmacokinetics of Bupleuri Radix: A Systematic Review. Biomed. Pharmacother. 131, 110679. 10.1016/j.biopha.2020.110679 32858498

[B15] Law K. P. Lim Y. P. (2013). Recent Advances in Mass Spectrometry: Data Independent Analysis and Hyper Reaction Monitoring. Expert Rev. Proteom. 10 (6), 551–566. 10.1586/14789450.2013.858022 24206228

[B16] Li D.-Q. Wu J. Liu L.-Y. Wu Y.-Y. Li L.-Z. Huang X.-X. (2015). Cytotoxic Triterpenoid Glycosides (Saikosaponins) from the Roots of Bupleurum Chinense. Bioorg. Med. Chem. Lett. 25 (18), 3887–3892. 10.1016/j.bmcl.2015.07.053 26259802

[B17] Li X. Li X. Huang N. Liu R. Sun R. (2018). A Comprehensive Review and Perspectives on Pharmacology and Toxicology of Saikosaponins. Phytomedicine 50, 73–87. 10.1016/j.phymed.2018.09.174 30466994 PMC7126585

[B18] Liang Z. Oh K. Wang Y. Yi T. Chen H. Zhao Z. (2014). Cell Type-specific Qualitative and Quantitative Analysis of Saikosaponins in Three Bupleurum Species Using Laser Microdissection and Liquid Chromatography-Quadrupole/time of Flight-Mass Spectrometry. J. Pharm. Biomed. Anal. 97, 157–165. 10.1016/j.jpba.2014.04.033 24863374

[B19] Liang Z. Zhang J. Yang G. Chen H. Zhao Z. (2013). Chemical Profiling and Histochemical Analysis of Bupleurum Marginatum Roots from Different Growing Areas of Hubei Province. Acta Pharm. Sin. B 3 (3), 193–204. 10.1016/j.apsb.2013.04.002

[B20] Liu G. Zhang Z. Lv X. Zhan S. Ding B. Yang X. (2019). Localization of Malonyl and Acetyl on Substituted Saikosaponins According to the Full‐scan Mass Spectra and the Fragmentation of Sodium‐adduct Ions in the Positive Mode. Rapid Commun. Mass. Spectrom. 33 (9), 883–893. 10.1002/rcm.8415 30771236

[B21] Luo S. Jin H. (1991). Studies on Chemical Constituents of Aboveground Parts of Six Medicinal Bupleurum Plants in Southwest China. Chin. Med. Mat 16 (6), 353–383. 1786097

[B22] Miyase T. Matsushima Y. (1997). Saikosaponin Homologues from Clinopodium Spp. The Structures of Clinoposaponins XII-XX. Chem. Pharm. Bull. 45 (9), 1493–1497. 10.1248/cpb.45.1493 9332001

[B23] Münger L. H. Boulos S. Nyström L. (2018). UPLC-MS/MS Based Identification of Dietary Steryl Glucosides by Investigation of Corresponding Free Sterols. Front. Chem. 6, 342. 10.3389/fchem.2018.00342 30186828 PMC6113793

[B24] Qing Z.-X. Zhao H. Tang Q. Mo C.-m. Huang P. Cheng P. (2017). Systematic Identification of Flavonols, Flavonol Glycosides, Triterpene and Siraitic Acid Glycosides from Siraitia Grosvenorii Using High-Performance Liquid Chromatography/quadrupole-Time-Of-Flight Mass Spectrometry Combined with a Screening Strategy. J. Pharm. Biomed. Anal. 138, 240–248. 10.1016/j.jpba.2017.01.059 28226282

[B25] Ren M. McGowan E. Li Y. Zhu X. Lu X. Zhu Z. (2019). Saikosaponin-d Suppresses COX2 through p-STAT3/C/EBPβ Signaling Pathway in Liver Cancer: A Novel Mechanism of Action. Front. Pharmacol. 10, 623. 10.3389/fphar.2019.00623 31191326 PMC6549044

[B26] Sánchez-Contreras S. Díaz-Lanza A. M. Bernabé M. (2000). Four New Triterpenoid Saponins from the Roots ofBupleurumrigidum. J. Nat. Prod. 63 (11), 1479–1482. 10.1021/np000004h 11087587

[B27] Shan L. Yang N. Zhao Y. Sheng X. Yang S. Li Y. (2018). A Rapid Classification and Identification Method Applied to the Analysis of Glycosides in Bupleuri Radix and Liquorice by Ultra High Performance Liquid Chromatography Coupled with Quadrupole Time-Of-Flight Mass Spectrometry. J. Sep. Sci. 41 (19), 3791–3805. 10.1002/jssc.201800619 30074686

[B28] Sinha S. K. Shakya A. Prasad S. K. Singh S. Gurav N. S. Prasad R. S. (2021). An *In-Silico* Evaluation of Different Saikosaponins for Their Potency against SARS-CoV-2 Using NSP15 and Fusion Spike Glycoprotein as Targets. J. Biomol. Struct. Dyn. 39 (9), 1–12. 10.1080/07391102.2020.1762741 32345124 PMC7232888

[B29] Sun P. Li Y. Wei S. Zhao T. Wang Y. Song C. (2018). Pharmacological Effects and Chemical Constituents of Bupleurum. Mrmc 19 (1), 34–55. 10.2174/1871520618666180628155931 29956627

[B30] Wang H. Feng M. L. Zhang Y. Xi X. H. Zhang X. H. Song M. Q. (2020). Comparative Study on Acute Toxicity, Antipyretic and Anti-inflammatory Effects of Bupleurum MarginatumVar.Stenophyllum and Bupleurum. Modernization Tradit. Chin. Med. Materia Med. World Sci. Techn. 22 (05), 1517–1523. 10.11842/wst.20190512001

[B31] Wang H. Feng M. L. Zhang Y. Xi X. H. Zhang X. H. Song M. Q. (2019). Protective Effect of Vinegar-Made Bupleurum Marginatum Var. Stenophyllum on Acute Liver Injury Induced by CCl4 in Mice. Cent. South Pharm. 10 (17), 1637–1641. 10.7539/j.issn.1672-2981.2019.10.010

[B32] Wang X. Wang Q. Burczynski F. J. Kong W. Gong Y. (2013). Saikosaponin A of Bupleurum Chinense (Chaihu) Elevates Bone Morphogenetic Protein 4 (BMP-4) during Hepatic Stellate Cell Activation. Phytomedicine 20 (14), 1330–1335. 10.1016/j.phymed.2013.07.010 23969230

[B33] Wang Z. Liu C.-H. Huang S. Chen J. (2019). Wnt Signaling in Vascular Eye Diseases. Prog. Retin. Eye Res. 70 (20), 110–133. 10.1016/j.preteyeres.2018.11.008 30513356 PMC6545170

[B34] Xia Z. Liu X. Tong L. Wang H. Feng M. Xi X. (2021). Comparison of Chemical Constituents of Bupleurum Marginatum Var. Stenophyllum and Bupleurum Chinense DC. Using UHPLC-Q‐TOF-MS Based on a Metabonomics Approach. Biomed. Chromatogr. 35 (9), e5133. 10.1002/bmc.5133 33811357

[B35] Xiao W. Wang Y. Zhang P. Li N. Jiang S. Wang J.-h. (2013). Bioactive Barrigenol Type Triterpenoids from the Leaves of Xanthoceras Sorbifolia Bunge. Eur. J. Med. Chem. 60, 263–270. 10.1016/j.ejmech.2012.12.022 23313635

[B36] Yang J.-B. Liu Y. Wang Q. Ma S.-C. Wang A.-G. Cheng X.-L. (2019). Characterization and Identification of the Chemical Constituents of Polygonum Multiflorum Thunb. By High-Performance Liquid Chromatography Coupled with Ultraviolet Detection and Linear Ion Trap FT-ICR Hybrid Mass Spectrometry. J. Pharm. Biomed. Anal. 172, 149–166. 10.1016/j.jpba.2019.03.049 31048141

[B37] Ye Y. H. Shi Y. Zhang B. W. Chen W. B. Ma Y. N. Yu H. (2019). Fingerprint Analysis of Bupleurum Chinense Roots from Different Origins by UPLC/Q-TOF-MS. Chin. J. Exp. Tradit Med. Form 25 (18), 124–129. 10.13422/j.cnki.syfjx.20190748

[B38] Yoshikawa I. M. (1997). New Hepatoprotective Saponins, Bupleurosides Iii, Vi, Ix, and Xiii, from Chinese Bupleuri Radix: Structure-Requirements for the Cytoprotective Activity in Primary Cultured Rat Hepatocytes. Bioorg. Med. Chem. Lett. 7, 2193. 10.1016/S0960-894X(97)00418-6

[B39] Yu J.-Q. Deng A.-J. Qin H.-L. (2014). Distinctive Features of Chemical Composition of Bupleurum Chinense Applicable to Original Authentication. Anal. Methods 25 (18), 1067–1075. 10.1039/c3ay41530a

[B40] Yu J.-Q. Deng A.-J. Wu L.-Q. Zhang Z.-H. Liu Y. Wang W.-J. (2013). Osteoclast-inhibiting Saikosaponin Derivatives from Bupleurum Chinense. Fitoterapia 85, 101–108. 10.1016/j.fitote.2013.01.005 23333582

[B41] Zhao X. Liu J. Ge S. Chen C. Li S. Wu X. (2019). Saikosaponin A Inhibits Breast Cancer by Regulating Th1/Th2 Balance. Front. Pharmacol. 10, 624. 10.3389/fphar.2019.00624 31214035 PMC6558179

[B42] Zhao Y.-y. Luo H.-s. Wang B. Ma L.-b. Tu G.-z. Zhang R.-y. (1996). Triterpenoid Saponins from Bupleurum Smithii Var. Parvifolium. Phytochemistry 42 (6), 1673–1675. 10.1016/0031-9422(96)00026-x 8783839

[B43] Zhu Y.-D. Hong J.-Y. Bao F.-D. Xing N. Wang L.-T. Sun Z.-H. (2018). Triterpenoid Saponins from Clinopodium Chinense (Benth.) O. Kuntze and Their Biological Activity. Arch. Pharm. Res. 41 (12), 1117–1130. 10.1007/s12272-017-0943-9 28895057

