# Corrigendum: Systematic Characterization and Identification of Saikosaponins in Extracts From *Bupleurum marginatum* var. *stenophyllum* Using UPLC-PDA-Q/TOF-MS

**DOI:** 10.3389/fchem.2022.867617

**Published:** 2022-04-21

**Authors:** Wenxi Liu, Xianlong Cheng, Rong Kang, Yadan Wang, Xiaohan Guo, Wenguang Jing, Feng Wei, Shuangcheng Ma

**Affiliations:** ^1^ Chinese Academy of Medical Science and Peking Union Medical College, Beijing, China; ^2^ National Institutes for Food and Drug Control, National Medical Products Administration, Beijing, China

**Keywords:** saikosaponins, radix bupleuri, UPLC-PDA-Q/TOF-MS, *Bupleurum marginatum* var. *stenophyllum*, *Bupleurum chinense* DC, *Bupleurum marginatum* Wall.ex DC

In the original article, there was a mistake in Supplementary Figure S45C as published. The panel erroneously included peak 83. The corrected figure is available in Supplementary Material.

In the original article, there was an error in listing some of the compounds in the **Results and Discussion** section, which were erroneously included in this section. A correction has been made in **Results and Discussion**, “*UPLC-PDA-Q/TOF-MS Analysis of the Saikosaponins Extract of BC and BMW*”:

“The same sample processing and UPLC-PDA-Q/TOF-MS detection methods were applied to identify saikosaponins of BC and BMW, which were then compared with BMS, to explore the similarities and differences of saikosaponins from three *Bupleurum* species. As shown in Supplementary Figure S45, except for a few individual saikosaponins (compounds 15 and 96 were not found in BC, compounds 15, 83 and 96 were not found in BMW), the saikosaponins in BMS were almost the same as those in BC and BMW, which have a material basis as an alternative variety of BC and BMW.”

The authors apologize for this error and state that this does not change the scientific conclusions of the article in any way. The original article has been updated.

