# Supplementary material for: Systematic Characterization and Identification of Saikosaponins in Extracts From Bupleurum marginatum var. stenophyllum Using UPLC-PDA-Q/TOF-MS
Source: Front Chem. 2021 Sep 30;9:747987. doi: 10.3389/fchem.2021.747987 (PMC8514958; doi:10.3389/fchem.2021.747987)
Supplement: Supplementary file 1 [file DataSheet1.docx]

**Supplementary materials**

**(Supplemental Tables and Figures)**

**Supplemental Table S1**

Relevant information of saikosaponin standard substances in ESI^−^

| name | RT  (min) | UV λmax (nm) | Molecular  Formula | Theoretical [M-H]^-^ | Experimental [M-H]^-^ | Error (ppm) | MS/MS |
| --- | --- | --- | --- | --- | --- | --- | --- |
| Tibesaikosaponin V | 20.13 | 210 | C_42_H_68_O_15_ | 811.4480 | 811.4507 | 3.33 | 781.4436,649.3923,619.3904,601.369,573.3837 ,503.3443,473.3309,455.3125,423.2911 |
| 11α-methoxy-SSf | 22.47 | 220 | C_49_H_82_O_18_ | 957.5423 | 957.5463 | 4.18 | 811.4909,795.4867 649.4308,617.4086,487.3802, 455.3471,407.3291, |
| Ne-SSk | 23.09 | 218 | C_48_H_80_O_18_ | 943.5266 | 943.5265 | -0.11 | 797.4672,781.468,635.4139,543.3696,473.3659,441.333 |
| SSc | 27.37 | 213 | C_48_H_78_O_17_ | 925.5161 | 925.5175 | 1.51 | 779.4551,763.4697,617.4031, 455.3498 |
| SSi | 27.43 | 281 | C_48_H_78_O_17_ | 925.5161 | 925.5175 | 1.51 | 779.4551,763.4697,617.4031, 455.3498,437.3433 ,407.3291 |
| SSf | 29.15 | 218 | C_48_H_80_O_17_ | 927.5317 | 927.5311 | -0.65 | 781.4718,765.4779,619.3904 ,457.3714 |
| SSh | 31.91 | 250 | C_48_H_78_O_17_ | 925.5161 | 925.5175 | 1.51 | 779.4551,763.4697,617.4031, 455.3498,407.3291 |
| SSb_3_ | 34.05 | 218 | C_43_H_72_O_14_ | 811.4844 | 811.4874 | 3.70 | 649.4308,573.3790 ,503.3737,471.3444,439.3239, 423.3271,391.2986 |
| SSb_4_ | 35.03 | 215 | C_43_H_72_O_14_ | 811.4844 | 811.4874 | 3.70 | 649.4308,573.3790 ,503.3737,471.3444,439.3239,423.2911,391.2986 |
| SSa | 37.96 | 219 | C_42_H_68_O_13_ | 779.4582 | 779.4590 | 1.03 | 617.4031,541.3459,471.3474,439.3172 |
| SSb_2_ | 38.39 | 251 | C_42_H_68_O_13_ | 779.4582 | 779.4590 | 1.03 | 617.4031,541.3459,471.3474,439.3172, 423.2911 |
| 6''-*O*-acetyl-SSb_3_ | 39.43 | 212 | C_45_H_74_O_15_ | 853.4949 | 853.4931 | -2.11 | 811.4909,793.4805,649.4308,573.3790,503.3737,471.3444,439.3239,423.3271, |
| 2''-*O*-acetyl-SSa | 39.50 | 219 | C_44_H_70_O_14_ | 821.4687 | 821.4728 | 4.99 | 779.4673,761.5461,617.4031,541.3459,471.3474,439.3172 |
| SSg | 39.94 | 281 | C_42_H_68_O_13_ | 779.4582 | 779.4590 | 1.03 | 617.4031,541.3560,471.3474,453.3371,439.3172, 423.2911 |
| Prosaikogenin f | 40.25 | 210 | C_36_H_58_O_8_ | 617.4053 | 617.4031 | -3.56 | 541.3459,471.3474,439.3172 |
| SSb_1_ | 40.47 | 250 | C_42_H_68_O_13_ | 779.4582 | 779.4590 | 1.03 | 617.4031,541.3459,471.3474,439.3172, 423.2911 |
| Prosaikogenin d | 40.56 | 251 | C_36_H_58_O_8_ | 617.4053 | 617.4031 | -3.56 | 541.3459,471.3474,439.3172, 423.2911 |
| SSe | 41.48 | 220 | C_42_H_68_O_12_ | 763.4633 | 763.4617 | -2.10 | 601.4063,525.352,455.3498 |
| 6''-*O*-acetyl-SSa | 42.61 | 210 | C_44_H_70_O_14_ | 821.4687 | 821.4728 | 4.99 | 779.4673,761.5461,617.4031,541.3459,471.3474,439.3172 |
| Prosaikogenin a | 43.01 | 251 | C_36_H_58_O_8_ | 617.4053 | 617.4031 | -3.56 | 541.3459,471.3474,439.3172, 423.2911 |
| SSd | 44.57 | 210 | C_42_H_68_O_13_ | 779.4582 | 779.4590 | 1.03 | 617.4031,541.3459,471.3474,439.3172 |
| 23-Hydroxy-13β,28β-epoxyolean-11-ene-16-one 3-*O*-β-D-glucopyranosyl-(1→3)-β-D-fucopyranoside | 45.93 | 218 | C_42_H_66_O_13_ | 777.4425 | 777.4449 | 3.09 | 615.3921 ,539.3361,469.330,437.3059 ,407.2939 |
| Prosaikogenin g | 49.33 | 210 | C_36_H_58_O_8_ | 617.4053 | 617.4031 | -3.56 | 541.3459,471.3474,439.3172 |
| 6''-*O*-acetyl-SSd | 53.31 | 210 | C_44_H_70_O_14_ | 821.4687 | 821.4728 | 4.99 | 779.4673,761.5461,617.4031,541.3459,471.3474 ,439.3172 |

(SS= saikosaponin)

**Supplemental Table S2** Mass spectrum information of related acetylated saikosaponin standard substance in the positive ion mode(ESI^+^)

| Name | MS  [M+Na]^+^ | [M+H]^+^ | MS/MS |
| --- | --- | --- | --- |
| 6''-*O*-acetyl-SSb_3_ | 877.4971 | 855.5052 | 823.4916,805.4705,455.3488,437.3436,419.3352 |
| 2''-*O*- acetyl-SSa | 845.4640 | 823.4916 | 823.4916,805.4705,787.4649,455.3488, 419.3352 |
| 6''-*O*-acetyl-SSa | 845.4640 | 823.4916 | 823.4916,805.4705,455.3488,437.3436,419.3352 |
| 6''-*O*-acetyl-SSd | 845.4640 | 823.4916 | 823.4916,805.4705 455.3488,437.3436；419.3352 |

(SS= saikosaponin)

**Supplemental Table S3**

Mass spectrum information of **acetylate/malonylated saikosaponins** in the positive ion mode (ESI^+^)

| No. | Identification | RT (min) | Experimental [M+H]^+^ | Experimental [M+Na]^+^ | MS/MS |
| --- | --- | --- | --- | --- | --- |
| 18 | *O*-acetyl- Bupleuroside V | 18.63 | 853.4697 | 875.4428 | 837.4761,485.3277,467.3181,449.3067 |
| 36 | *O*-acetyl-SSs | 22.78 | 985.5385 | 1007.5225 | 455.3488,437.3436,419.3352 |
| 39 | *O*-acetyl-SSs | 23.6 | 985.5385 | 1007.5225 | 455.3488,437.3436,419.3352 |
| 43 | *O*-malony- hydroxysaikosaponin c | 24.94 | 1031.5449 |  | 457.3718,439.3537,421.3483 |
| 46 | *O*-acetyl-SSs | 26.66 | 985.5385 | 1007.5225 | 455.3488,437.3436,419.3352 |
| 53 | 2'''-*O*- acetyl- SSc | 30.06 | 969.5455 | 991.5323 | 439.3537,421.3483,403.3338 |
| 55 | 3'''-*O*- acetyl- SSc | 30.81 | 969.5455 | 991.5323 | 439.3537,421.3483,403.3338 |
| 57 | 2'''-*O*- acetyl- SSf | 32.58 | 971.5528 | 993.5339 | 441.3776,423.3662,405.3521 |
| 59 | 3'''-*O*- acetyl- SSf | 32.97 | 971.5528 | 993.5339 | 441.3776,423.3662,405.3521 |
| 61 | 23-*O*-acetyl-(11α-methoxyl-SSs) | 33.51 |  | 1039.5776 | 529.4238,511.4151 |
| 62 | 4'''-*O*- acetyl- SSc | 33.72 | 969.5455 | 991.5323 | 439.3537,421.3483,403.3338 |
| 63 | 4'''-*O*- acetyl- SSf | 33.79 | 971.5528 | 993.5339 | 441.3776,423.3662,405.3521 |
| 66 | 6'''-*O*- acetyl- SSc | 35.49 | 969.5455 | 991.5323 | 439.3537,421.3483,403.3338 |
| 67 | 2''-*O*- acetyl- SSb_3_ | 35.62 | 855.5052 | 877.4971 | 823.4916,805.4705,455.3488,437.3436,419.3352 |
| 69 | 6'''-*O*- acetyl- SSf | 36.29 | 971.5528 | 993.5339 | 441.3776,423.3662,405.3521 |
| 70 | *O*-acetyl-SS b_3_/b_4_ | 36.97 | 855.5052 | 877.4971 | 823.4916,805.4705,455.3488,437.3436,419.3352 |
| 78 | 2''-*O*- acetyl- SSb_2_ | 39.14 | 823.4916, | 845.4640 | 823.4916,805.4705, 455.3488,437.3436,419.3352 |
| 80 | 6''-*O*- acetyl- SSb_3_ | 39.43 | 855.5052 | 877.4971 | 823.4916,805.4705,455.3488,437.3436,419.3352 |
| 81 | 2''-*O*- acetyl- SSa | 39.50 | 823.4916, | 845.4640 | 823.4916,805.4705, 455.3488,437.3436,419.3352 |
| 82 | *O*- malonyl- SSa | 39.68 | 867.4714 | 889.4564 | 455.3488,437.3436,419.3352 |
| 83 | 3''-*O*- acetyl- SSb_2_ | 39.68 | 855.5052 | 877.4971 | 823.4916,805.4705,455.3488,437.3436,419.3352 |
| 85 | 6''-*O*- acetyl- SSb_4_ | 40.12 | 855.5052 | 877.4971 | 823.4916,805.4705,455.3488,437.3436,419.3352 |
| 87 | 3''-*O*- acetyl- SSa | 40.29 | 855.5052 | 877.4971 | 823.4916,805.4705,455.3488,437.3436,419.3352 |
| 88 | 4''-*O*- acetyl- SSa | 40.42 | 823.4916 | 845.4640 | 823.4916,805.4705 455.3488,437.3436,419.3352 |
| 91 | *O*- malonyl-acetyl-SSa | 41.74 | 909.4878 | 931.4720 | 455.3488,437.3436,419.3352 |
| 92 | 6''-*O*- acetyl- SSa | 42.61 | 823.4916 | 845.4640 | 823.4916,805.4705, 455.3488,437.3436,419.3352 |
| 93 | O-acetyl-SSb_1_ | 42.87 | 823.4916 | 845.4640 | 823.4916,805.4705, 455.3488,437.3436,419.3352 |
| 98 | 4''-*O*- acetyl- SSb_2_ | 45.05 | 823.4916 | 845.4640 | 823.4916,805.4705 455.3488,437.3436,419.3352 |
| 100 | 6''-*O*- acetyl- SSb_2_ | 45.45 | 823.4916 | 845.4640 | 823.4916,805.4705 455.3488,437.3436,419.3352 |
| 101 | *O*- malonyl- SSd | 47.22 | 867.4714 | 889.4564 | 455.3488,437.3436,419.3352 |
| 102 | 2''-*O*- acetyl- SSa | 47.83 | 823.4916 | 845.4640 | 823.4916,805.4705,455.3488,437.3436,419.3352 |
| 103 | 3''-*O*- acetyl- SSa | 48.01 | 855.5052 | 877.4971 | 823.4916,805.4705,455.3488,437.3436,419.3352 |
| 104 | 4''-*O*- acetyl- SSa | 48.58 | 823.4916 | 845.4640 | 823.4916,805.4705,455.3488,437.3436,419.3352 |
| 106 | *O*- diacetyl - SSd | 51.80 |  | 887.4792 | 455.3488,437.3436,419.3352 |
| 107 | *O*- malonyl-acetyl-SSd | 51.80 |  | 931.4720 | 455.3488,437.3436,419.3352 |
| 108 | *O*- diacetyl - SSd | 32.90 |  | 887.4792 | 455.3488,437.3436,419.3352 |
| 109 | 6''-*O*- acetyl- SSd | 53.31 | 823.4916 | 845.4640 | 823.4916,805.4705 ,455.3488,437.3436,419.3352 |


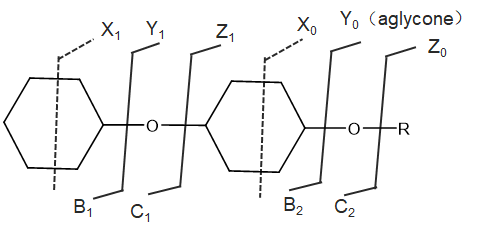


**Supplemental Figure S1** fragmentation pathway of their saikosaponins according to the structural nomenclature of Domon and Costello.





**Supplemental Figure S2** The fragmentation pathway of 23-Hydroxy-13β,28β-epoxyolean-11-ene-16-one 3-*O*-β-D-glucopyranosyl-(1→3)-β-D-fucopyranoside


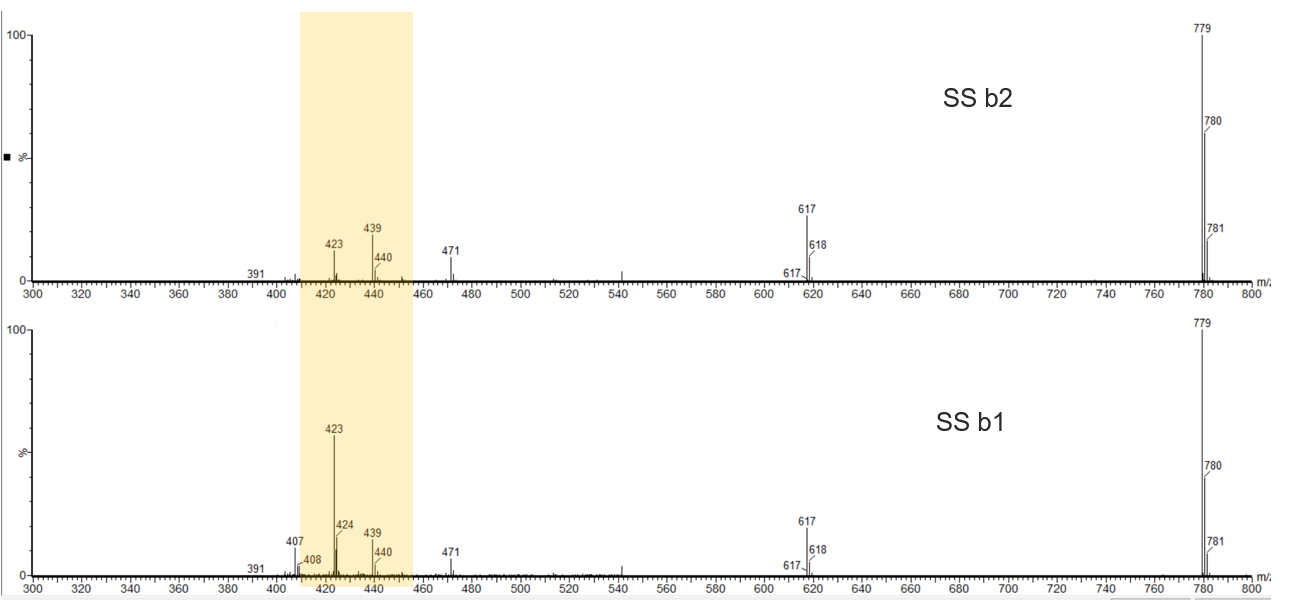


**Supplemental Figure S3**

The MS/MS spectrum of SSb_1_ and SSb_2_





**Supplemental Figure S4** The fragmentation pathway of SSh





**Supplemental Figure S5** The fragmentation pathway of SSf





**Supplemental Figure S6** The fragmentation pathway of Ne-SSk



**Supplemental Figure S7**

The fragmentation pathway of 11α-methoxy-SSf





**Supplemental Figure S8**

The fragmentation pathway of 6''-*O*-acetyl-SSa





**Supplemental Figure S9**

The fragmentation pathway of 6''-*O*- malonyl -SSa





**Supplemental Figure S10** The fragmentation pathway of 6''-*O*-acetyl-SSb_3_ in positive ion mode (ESI^+^)





**Supplemental Figure S11**

The fragmentation pathway of 23-*O*-acetyl-SSa in positive ion mode(ESI^+^)





**Supplemental Figure S12**

The fragmentation pathway of Clinoposaponin XIV(compound 42)



**Supplemental Figure S13**

The structures of compounds **40**,**58**,**71**



**Supplemental Figure S14**

The fragmentation pathway of SSs / SSn(compounds 31 and 33)





**Supplemental Figure S15**

The fragmentation pathway of SSl /Tibesaikosaponin IV(compounds 13 and 38)





**Supplemental Figure S16**

The fragmentation pathway of SSq(compounds 2 ,14)





**Supplemental Figure S17** The structures of compounds **7**,**50** and **76**





**Supplemental Figure S18** The fragmentation pathway of Tibesaikosaponin II (compounds 17 and 77)





**Supplemental Figure S19**

The structure of 3β,​23,​28-​Trihydroxyolean-​11,​13(18)​-​diene-​16-​one 3-*​O*-​β-​D-​glucopyranosyl-​(1-3)​-​β-​D-​fucopyranoside (compound 79)





**Supplemental Figure S20**

The fragmentation pathway of SSt (compound 8)





**Supplemental Figure S21**

The Structure of SSt(Bupleuroside VI,compound 30)





**Supplemental Figure S22**

The fragmentation pathway of Rotundioside P (compound 3)





**Supplemental Figure S23**

The structures of hydroxysaikosaponin a, hydroxysaikosaponin d(compound 25 and 27)





**Supplemental** **Figure S24**

The fragmentation pathway of 3β, 16α, 23,28-Tetrahydroxy-olean-9,12(13)-dien-29-oic acid 3-O-β-D-glucopyranosyl-(1-3)-β-D-fucopyranoside (compound 6)





**Supplemental Figure S25**

The fragmentation pathway of Bupleuroside V (compound 10)





**Supplemental Figure S26**

The structure of 3β,16α,23,28-Tetrahydroxy-olean-11,13(18)-dien-30-oicacid3-O-β-D-glucopyranosyl-(1-2)-β-D-glucopyranosyl-(1-3)-β-D-fucopyranoside(compound 9)





**Supplemental Figure S27**

The fragmentation pathway of hydroxysaikosaponin t (compound 1)





**Supplemental Figure S28**

The structures of hydroxysaikosaponin c ,hydroxysaikosaponin h and hydroxysaikosaponin b_2_





**Supplemental Figure S29** The fragmentation pathway of 11α-ethoxyl-SSs (compound 32)





**Supplemental Figure S30**

The fragmentation pathway of 11α -ethoxyl-SSb_1_/11α-ethoxyl-SSb_2_(compound 72/74)





**Supplemental Figure S31**

The fragmentation pathway of 11α- butoxyl-SSb_2_ (compound 96)





**Supplemental Figure S32**

The fragmentation pathway of 11α-ethoxyl-SSh (compound 45)





**Supplemental Figure S33**

The possible structures of dihydro-SSq and its isomers(Compounds 4,12,16,19 and 20)





**Supplemental Figure** **S34**

The structure of Δ^21,22^-Tibesaikosaponin I(compound 41)





**Supplemental Figure S35**

The fragmentation pathway of Tibesaikosaponin I (compound 29 and 94)





**Supplemental Figure S36**

The fragmentation pathway of Magnoside B (compound 21)





**Supplemental Figure S37** The structures of compounds 22,5,15,68 and 95





**Supplemental Figure S38** The structures of acetylated derivatives of SSa, SSd and SSb_2_





**Supplemental Figure S39**

The fragmentation pathway of 6'''-*O*- acetyl- SSc in the negative ion mode





**Supplemental Figure S40** The structures of acetylated derivatives of SSc/SSf





**Supplemental Figure S41**

The structures of compound **67,80** and **85**





**Supplemental Figure S42**

The structures of 2'''- *O*-acetyl-SSs、3'''- *O*-acetyl-SSs、4'''- *O*-acetyl-SSs、6'''- *O*-acetyl-SSs





**Supplemental Figure S43**

The fragmentation pathway of 6'''-*O*-acetyl-SSs in the negative ion mode





**Supplemental Figure S44**

The fragmentation pathway of 23-*O*-acetyl-(11α-methoxyl-SSs) in negative ion mode


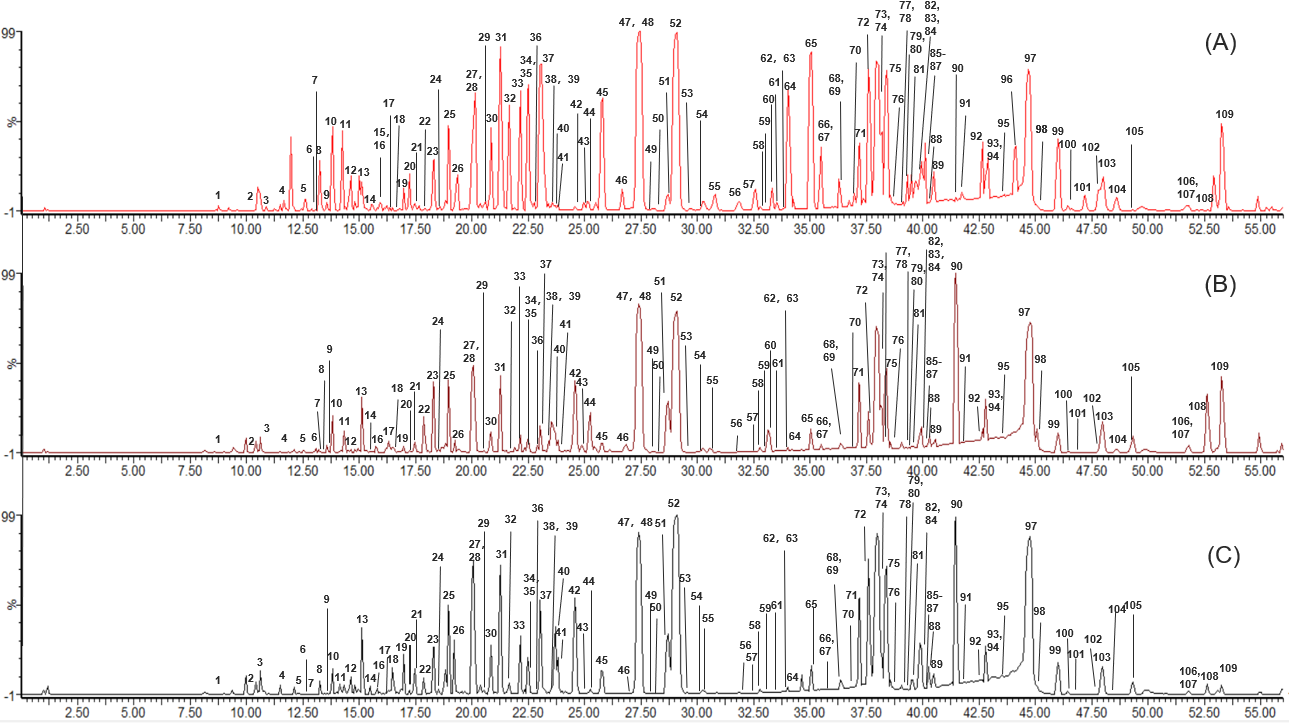


**Supplemental Figure S45**

Base peak ion chromatograms of *Bupleurum marginatum var. stenophyllum* (A), *Bupleurum chinense DC.* (B) and *Bupleurum marginatum Wall.ex DC.* (C) in ESI^–^
